# Total Synthesis and Stereochemical Assignment of Nostosin B

**DOI:** 10.3390/md15030058

**Published:** 2017-02-27

**Authors:** Xiaoji Wang, Junmin Feng, Zhengshuang Xu, Tao Ye, Yi Meng, Zhiyu Zhang

**Affiliations:** 1 School of Life Science, Jiangxi Science and Technology Normal University, Nanchang 330013, China; vonjimi@gmail.com; 2 Laboratory of Chemical Genomics, Engineering Laboratory for Chiral Drug Synthesis, School of Chemical Biology and Biotechnology, Peking University Shenzhen Graduate School, University Town of Shenzhen, Xili, Nanshan District, Shenzhen 518055, China; mengy@pkusz.edu.cn; 3 State Intellectual Property Office of the People's Republic of China, Kaiyuan Road, Guang Zhou 510000, China; 13246888828@163.com

**Keywords:** nostosin B, total synthesis, stereochemical assignment

## Abstract

Nostosins A and B were isolated from a hydrophilic extract of *Nostoc* sp. strain from Iran, which exhibits excellent trypsin inhibitory activity. Nostosin A was the most potent natural tripeptide aldehyde as trypsin inhibitor up to now. Both *r*- and *s*-2-hydroxy-4-(4-hydroxy-phenyl)butanoic acid (Hhpba) were prepared and incorporated into the total synthesis of nostosin B, respectively. Careful comparison of the NMR spectra and optical rotation data of synthetic nostosin B (**1a** and **1b**) with the natural product led to the unambiguous identification of the *r*-configuration of the Hhpba fragment, which was further confirmed by co-injection with the authentic sample on HPLC using both reversed phase column and the chiral AD-RH column.

## 1. Introduction

**Figure 1:**
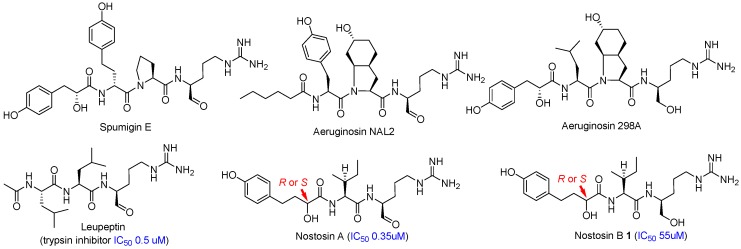
Natural peptide aldehyde and structure of nostosins A and B and leupeptin.

Nostosin A and B were isolated from a hydrophilic extract of *Nostoc* sp. strain obtained from a paddy field in Iran (Figure 1) [1]. Both nostosin A and B showed potent inhibitory activity toward porcine trypsin, with IC_50_ values of 0.35 and 55 μM respectively. Structurally and functionally, nostosin A and B could be classified as natural small linear peptides that act as trypsin inhibitors [2,3,4,5]. These small peptides structurally feature an arginine motif at the C-terminal, which has been proven to be crucial for their interactions with the protease targets [6,7,8,9,10,11]. Surprisingly, nostosin A was more potent than the commercially available trypsin inhibitor leupeptin (IC_50_ = 0.5 μM), rendering it as the most potent tripeptide natural trypsin inhibitor up to now.

Both nostosin A and B contain three subunits, 2-hydroxy-4-(4-hydroxyphenyl)butanoic acid (Hhpba), *l*-Ile and *l*-Arg, the C-terminal of nostosin B exists as the reduced form of nostosin A, which dramatically diminished its biological activity (158-fold compared to nostosin A), similar to aeruginosin 298 A [12,13,14]. The absolute stereochemistry of Hhpba was not elucidated [1]. Although the authors of isolation paper prefer that Hhpba possesses *r*-configuration according to the statistical data of the stereochemistry of similar residue of 39 trypsin inhibitors isolated from natural microorganisms, the docking results of both nostosins A and B with trypsin made no obvious difference whatever the *r*- or *s*-Hhpba was adopted [1].

Small peptide aldehydes had long been used as proteasome inhibitors, further development of these compounds as antibacterial, anticancer, or other therapeutic reagents have also been reported, e.g., fellutamide B was used to reveal the connection of proteasome inhibition and nerve growth factor (NGF) production, which could have potential for the treatment of neuronal injury and neurodegenerative diseases [15,16,17]. Herein, we report our total synthesis and stereochemical assignment of nostosin B.

## 2. Results

**Figure 2:**
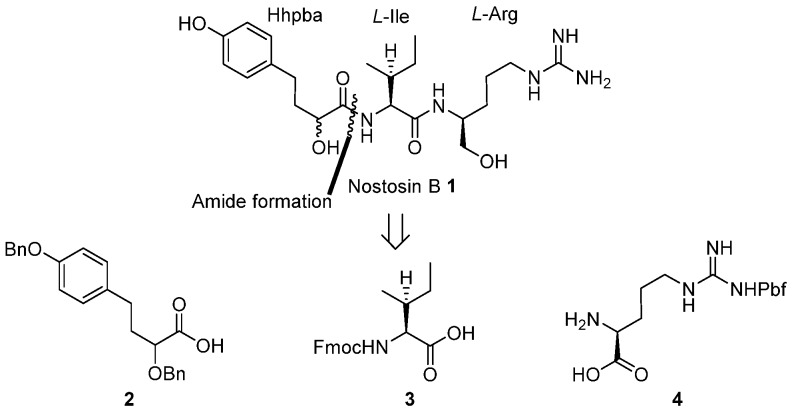
Retrosynthetic analysis of nostosin B **1**.

Nostosins A and B are distinctive trypsin inhibitors isolated from nature, however the C-2 stereochemistry of Hhpba has not been defined, which hampers the further development of nostosins as biological probes or lead candidates. As part of our research interests [18,19,20,21,22,23,24,25,26,27,28,29], we decided to verify the absolute stereochemistry of nostosins via the total synthesis of both stereoisomers of nostosin B **1**. Since the Hhpba fragment was located at the N-terminal of nostosin B, we decided to prepare this tripeptide via a 1 + 2 strategy, the late-stage introduction of Hhpba made the synthesis more convenient for the preparation of two stereoisomers (Figure 2). The arginine motif in nostosin B could be easily obtained from Pbf-*l*-Arg **4**.

### 2.1. Synthesis of the Right-Hand Dipeptide

**Scheme 1:**
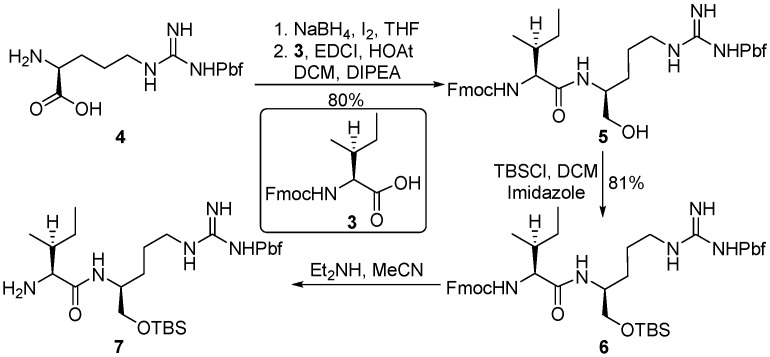
The synthesis of dipeptide **7**.

The synthesis was commenced from the reduction of the carboxylic group of *N*^ω^-Pbf-*l*-arginine **4**, the resulted amino alcohol was coupled with Fmoc-*l*-isoleucine **3**. The reaction was facilitated by 1-(3-dimethylaminopropyl)-3-ethylcarbodiimide hydrochloride and 1-hydroxy-7-azabenzotriazole in the presence of *N*,*N*-diisopropylethylamine in dichloromethane to produce dipeptide **5** in 80% yield over two steps. The primary alcohol of **5** was protected by *tert*-butyldimethylsilyl chloride to give silyl ether **6** in 81% yield. Removal of the fluorenyl-9-methoxycarbonyl group via treatment of **6** with diethylamine in acetonitrile provided amine **7**, which was ready for the next step of transformation (Scheme 1).

### 2.2. Synthesis of Hhpba Fragment

2-hydroxy-4-arylbutyrates, especially 2-hydroxy-4-phenylbutyrate, are key intermediates for the preparation of angiotensin-converting enzyme (ACE) inhibitors, upon transformation of the secondary alcohol to amino moiety [30]. Hence, several technical processes have been developed to produce the enantiomerically pure 2-hydroxy-4-phenylbutyrate, including (i) enzymatic resolution of the racemic 2-hydroxy-4-phenylbutyrate; (ii) asymmetric hydrogenation of 2-oxo-4-phenylbutyrate, 2,4-dioxo-4-phenylbutyrate, or 2-oxo-4-arybut-3-enoate in the presence of chiral catalysts [31,32]. The bio-processes for this important intermediate usually suffer low efficiency, whilst the asymmetric hydrogenations afford only moderate to good optical purity, let alone the catalysts or chiral ligands being difficult to obtain.

**Scheme 2:**
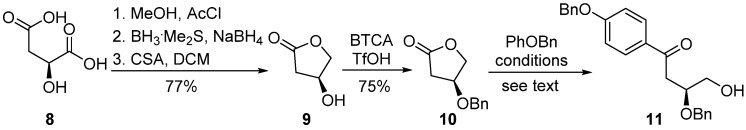
Attempt synthesis of Hhpba by Friedel-Crafts reaction.

To prepare the Hhpba fragment, we initially took advantage of malic acid via the Friedel-Crafts reaction [33], anticipating that both *r*- and *s*-Hhpba could be originated from *d*- and *l*-malic acid, respectively. According to literature precedent, both acetoxysuccinic anhydride and 2-acetoxy-butanoyl chloride had been used to react with benzene or methoxy-substituted benzenes, providing 2-hydroxy-4-aryl-4-oxobutanoates in moderate yields [34,35]. To eliminate the interference of the acetyl group on acetoxysuccinic anhydride in the process of Friedel-Crafts reaction, we decided to protect the hydroxyl group of **9** with benzyl group (Scheme 2).

Esterification of *l*-malic acid **8** was performed using standard conditions. Hydroxyl group directed regio-selective reduction of the alpha-methyl ester, followed by acid catalyzed cyclization produced lactone **9** at a 77% yield [36,37]. *O*-benzylation of the sensitive hydroxyl group of **9** was achieved using benzyl trichloroacetimidate (BTCA) in the presence of a catalytic amount of triflic acid [38], affording compound **10** at a 75% yield. The Friedel-Crafts reaction between **10** and benzyl phenyl ether was then performed under different reaction conditions (Table 1).

Using aluminum trichloride as Lewis acid, toluene as solvent, the Friedel-Crafts reaction was carried out either under reflux or at room temperature (entries 1 and 2), both conditions provided no desired product with only decomposition of starting material. When the reaction solvent was changed to dichloroethane (entries 3 and 4), or Lewis acid was changed to trifluoroborane diethyletherate (entry 5), there was still no desired product and the starting material was not recoverable. While molecular sieves (4 Å) did not destroy the substrate, nor did they promote the Friedel-Drafts reaction (entry 6).

**Scheme 3:**
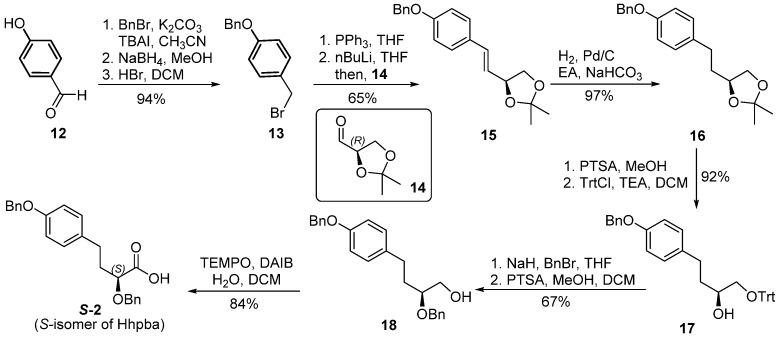
Stereocontrolled synthesis of *s*-Hhpba.

We then turned to develop an asymmetric strategy for Hhpba fragments using commercial available aldehyde **14** and **12** as starting material (Scheme 3). Thus, protection of vanillin **12** with benzylbromide in the presence of potassium carbonate and tetrabutylammonium iodide in acetonitrile, followed by reduction of the aldehyde with sodium borohydride in methanol and bromination of the corresponding benzyl alcohol with hydrogen bromide in dichloromethane, afforded compound **13** at a 94% yield over three steps. The ylide, generated by deprotonation with n-butyllithium of the intermediate by refluxing of **13** with triphenylphosphine, was reacted with aldehyde **14** to forge alkene **15** at a 65% yield [39]. Hydrogenation of **15** with palladium on charcoal in ethyl acetate in the presence of sodium bicarbonate smoothly saturated the double bond with the benzyl ether untouched, giving compound **16** at a 97% yield. The acetonide was subsequently removed using *p*-toluenesulfonic acid in methanol, the primary alcohol was masked using trityl chloride in dichloromethane in the presence of trimethylamine to afford the secondary alcohol **17** at a 92% yield over two steps. Protection of the free alcohol with benzyl bromide, followed by releasing the primary alcohol, afforded compound **18** at a 67% yield. The synthesis of Hhpba was accomplished via mild oxidation using 2,2,6,6-tetramethylpiperidoxyl, iodobenzene diacetate in water, and dichloromethane [40], producing ***S*-2** at an 84% yield.

**Scheme 4:**
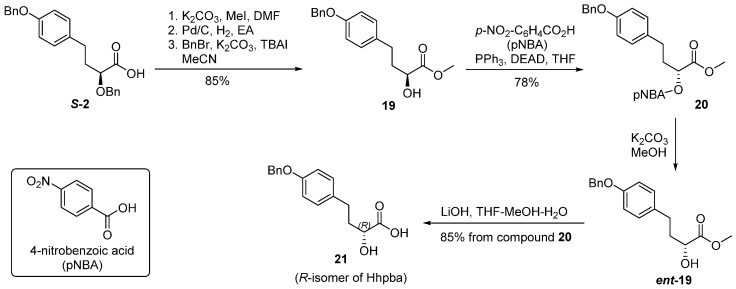
Preparation of *r*-Hhpba.

With ***S*-2** in hand, we set forth to prepare its *R*-enantiomer (Scheme 4). Treatment of acid ***S*-2** with iodomethane and potassium carbonate in *N*,*N*-dimethylformamide, followed by hydrogenation to remove the benzyl ethers and subsequent re-protection of the phenol as benzyl ether, produced methyl ester **19** at an 85% yield over three steps. Mitsunobu reaction of the secondary alcohol **19** with 4-nitrobenzoic acid (pNBA) in the presence of triphenylphosphine and diethyl azodicarboxylate in tetrahydrofuran gave ester **20** at a 78% yield. A two-step removal of the protection groups was performed at this stage, first treatment of diester **20** with potassium carbonate in methanol produced ***ent*-19**, which demonstrated the integrity of stereochemistry by comparison of the analytical data with compound **19**. Further hydrolysis of the methyl ester of ***ent-*19** gave compound **21** at an 85% overall yield.

### 2.3. Total Synthesis of Nostosin B

With both *s*- and *r*-Hhpba (***S***-**2** and **21**) in hand, we were ready to complete the total synthesis of nostosin B and confirm its absolute stereochemistry.

**Scheme 5:**
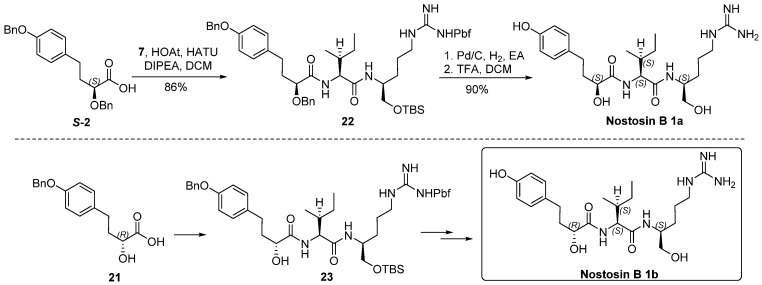
Completion of the total synthesis of nostosin B (both **1a** and **1b**).

As shown in Scheme 5, reaction of acid ***S*-2** and dipeptide **7**, using *O*-(7-azabenzotriazol-1-yl)-*N*,*N*,*N*,*N*-tetramethyl uronium hexafluorophosphate as dehydration reagent in the presence of 1-hydroxy-7-azabenzotriazole and *N,N*-diisopropylethylamine in dichloromethane, gave tripeptide **22** at an 86% yield. Catalytic hydrogenation removed the benzyl ethers, subsequent treatment with trifluroacetic acid cleaved simultaneously both 2,2,4,6,7-pentamethyl-2,3-dihydrobenzofuran-5-sulfonyl and *tert*-butylsilyl groups from the molecule, producing nostosin B **1a** at a 74% yield over two steps. Using similar strategy, the synthesis of nostosin B **1b** was accomplished starting from hydroxyl acid **21** at a 64% yield over three steps.

## 3. Discussion

The NMR spectra data for nostosin B **1a** and **1b** were very similar, but both were slightly different from the reported spectra of the natural product [1]. Nostosin B **1b** has the same sign on optical rotation ([α]${}_{D}^{20}$ = −2.4 (*c* 1.1, MeOH)) with natural product ([α]${}_{D}^{20}$ = −2.9 (*c* 0.08, H_2_O)), while nostosin B **1a** shows the opposite sign ([α]${}_{D}^{20}$ = 1.6 (*c* 1.0, MeOH)). From these analytical data, we believe that nostosin **1b** was the real structure of the natural product, the slight differences on NMR spectra should be arisen from the different experimental conditions for data acquisition, i.e., the concentration of sample and pH of the solution.

**Figure 3:**
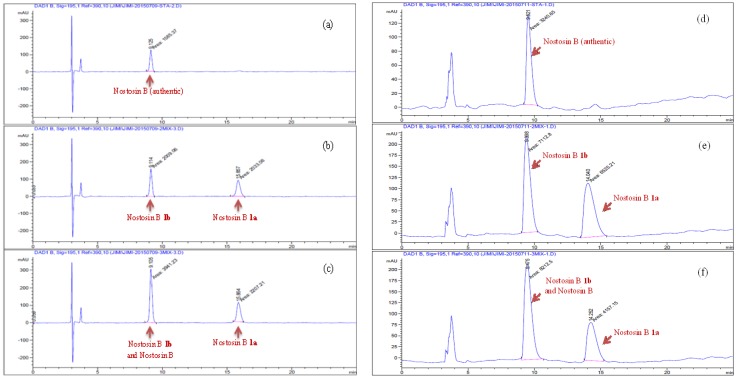
HPLC analyses of nostosin B samples (authentic sample (natural product) and the synthetic samples (**1a** and **1b**). **Left column**: Agilent 1200, XB-C18 column (Welch Materials. (Shanghai) Inc. Shanghai, China. 5 μm, 4.6 × 250 mm), eluting with acetonitrile and water (MeCN/H_2_O = 18/82, *v*/*v*; flow rate 1.0 mL/min). Diode array detector (DAD) was set to 195 nm^−1^ wave length. (**a**) Nostosin B (authentic sample); (**b**) Synthetic samples (Nostosin B **1a** and Nostosin B **1b**); (**c**) Co-injection of the authentic sample and synthetic samples; **Right column**: Agilent 1200, AD-RH column (Chiralpak, Daicel Chiral Technologies (China) Co., LTD. Shanghai, China. 5μm, 4.6 × 250 mm), eluting with acetonitrile and water (MeCN/H_2_O = 7/93, *v*/*v*; flow rate 0.5 mL/min). Diode array detector (DAD) was set to 195 nm^−1^ wave length; (**d**) Nostosin B (authentic sample); (**e**) Synthetic samples (Nostosin B **1a** and Nostosin B **1b**); (**f**) Co-injection of the authentic sample and synthetic samples.

To further identify the stereochemistry of nostosin B, the synthetic samples (**1a** and **1b**) were co-injected with the authentic sample (natural product sample) using high-performance liquid chromatography (Figure 3). Both reverse phase column and chiral column gave the same results, indicating that synthetic nostosin B **1b** has identical retention time with natural nostosin B, unambiguously defining the stereochemistry of Hhpba as *r*-configuration.

## 4. Materials and Methods

### 4.1. General Experiment

Non-aqueous reactions were performed under a nitrogen or argon atmosphere and all reaction vessels were oven-dried. Solvents were distilled prior to use: dichloromethane (DCM), triethylamine and diisopropylethylamine (DIPEA) from CaH_2_, tetrahydrofuran (THF) from Na/benzophenone. TLCs were carried out using pre-coated sheets (Qingdao silica gel 60-F_250_, 0.2 mm, Qingdao, China) and visualized at 254 nm, and stained in ninhydrin or phosphomolybdic acid solution followed by heating. Flash column chromatography was performed on E. Qingdao silica gel 60 (230–400 mesh ASTM) using the indicated solvents. Optical rotations were measured on AUTOPOL I automatic polarimeter (Rudolph Research Analytical, Hackettstown NJ 07840, USA). NMR spectra were recorded on Bruker spectrometers (Bruker BioSpin AG, Industriestrasse 26, 8117 Fällanden, Switzerland). Chemical shifts were reported in parts per million (ppm), relative to the signals of solvent residue. Data were reported as follows: chemical shift, multiplicity (s = singlet, d = doublet, t = triplet, q = quartet, br = broad), integration and coupling constant. ESI mass spectra were obtained using a AB QSTAR Elite mass spectrometer (International Equipment Trading Ltd. Mundelein, IL 60060, USA).

### 4.2. Synthesis of the Right-Hand Dipeptide Fragment

NaBH_4_ (2.13 g, 56.27 mmol) was suspended in THF (100 mL) at 0 °C, *l*-Pbf-Arg **4** (10.00 g, 23.45 mmol) was added in one portion. A solution of I_2_ (5.95 g, 23.45 mmol) in THF (20 mL) was dropwise added to the above amino acid solution within 0.5 h. After gas evolution ceased, the reaction mixture was heated to reflux for 18 h. The reaction temperature was cooled to room temperature, and methanol was carefully added to quench the reaction until the reaction solution became clear. Stir was continued for 0.5 h, the solution was concentrated in vacuo. The residue was dissolved by aqueous solution of KOH (50 mL, 20% in water) and stirred for 4 h, then the solution was extracted by CH_2_Cl_2_ (100 mL × 3). The combined organic layers were washed with water (50 mL), dried over anhydrous sodium sulfate and concentrated in vacuo to afford the amino alcohol (6.28 g, 65%) as crude oil.

To a solution of amino alcohol (0.58 g, 1.42 mmol) in CH_2_Cl_2_ (20 mL), Fmoc-Ile-OH **3** (0.50 g, 1.42 mmol), HOAt (0.29 g, 2.12 mmol), and EDCI (0.27 g, 1.42 mmol) were sequentially added at 0 °C, after DIPEA (0.47 mL, 2.83 mmol) was added via a syringe, the reaction mixture was brought to room temperature and stirred for 16 h. Volatiles were removed in vacuo. The residue was dissolved in ethyl acetate (150 mL) and washed with saturated aqueous solution of NH_4_Cl (30 mL), NaHCO_3_ (30 mL), and brine (30 mL). The organic phase was dried over anhydrous sodium sulfate and concentrated in vacuo. The residue was purified by flash chromatography to give dipeptide **5** (0.84 g, 80%) as yellow oil.

Compound **5** (0.53 g, 0.70 mmol) was dissolved in DCM (5 mL) at 0 °C, after imidazole (0.24 g, 3.5 mmol) and TBSCl (0.21 g, 1.40 mmol) were added, the reaction mixture was brought to room temperature and stirred for 16 h. The reaction mixture was diluted by DCM (50 mL), the organic solution was washed with saturated aqueous solution of NH_4_Cl (30 mL), brine (30 mL). The organic layer was dried over anhydrous sodium sulfate and concentrated in vacuo. The residue was purified by flash chromatography to afford compound **6** (0.49g, 81%). [α]${}_{D}^{20}$ = −28.7 (*c* 1.0, CH_2_Cl_2_); ^1^H-NMR (500 MHz, CDCl_3_) δ 7.72–7.70 (m, 2H), 7.53–7.51 (m, 2H), 7.37–7.32 (m, 2H), 7.25–7.22 (m, 2H), 6.45 (br, 1H), 6.13 (br, 2H), 5.63 (br, 1H), 4.41–4.37 (dd, *J* = 7.5, 10.5 Hz, 1H), 4.26–4.22 (m, 1H), 4.14–4.10 (m, 1H), 4.03–3.90 (m, 1H), 3.52 (s, 2H), 3.18 (br, 1H), 3.10 (br, 1H), 2.89 (s, 2H), 2.54 (s, 3H), 2.48 (s, 3H), 2.04 (s, 3H), 1.88–1.78 (m, 2H), 1.57–1.44 (m, 4H), 1.41 (s, 6H), 1.16–1.12 (m, 1H), 0.90–0.88 (m, 6H), 0.85 (s, 9H), 0.01 (s, 6H). ^13^C-NMR (125 MHz, CDCl_3_) δ 171.79, 158.74, 156.68, 156.22, 143.94, 143.69, 141.38, 138.45, 132.40, 127.82, 127.21, 125.11, 124.58, 120.07, 117.46, 88.34, 67.26, 65.02, 60.03, 47.22, 43.35, 41.22, 37.50, 29.15, 28.65, 25.94, 25.75, 25.33, 25.10, 19.30, 18.34, 17.95, 15.54, 12.50, 11.46, −5.42, −5.44 ppm; HRMS (ESI) calculated for C_17_H_20_O_3_ [M + Na]^+^ 884.4530, found 884.4434.

Compound **6** (55 mg, 0.064 mmol) was dissolved in CH_3_CN (2 mL) and cooled to 0 °C, after diethylamine (0.07 mL, 0.64 mmol) was added, the reaction mixture was brought to room temperature and monitored by TLC. Upon the consumption of all starting materials, the reaction mixture was concentrated in vacuo. The residue was dissolved in DCM (2 mL) and concentrated in vacuo, these procedures were repeated twice. The residue was dried under high vacuum for 1 h to give the crude amine **7**, which was used directly without further purification.

### 4.3. Synthesis of the Hhpba Fragment

#### 4.3.1. Synthesis of *s*-Hhpba (**S-2**)

Compound **15** [39] (3.55 g, 11.42 mmol) was dissovled in ethyl acetate (50 mL), Pd-C (355 mg, 10% on charcoal) and sodium bicarbonate (10.00 g, 114.24 mmol) were added. The reaction vessel was sealed and the atmosphere was changed to hydrogen. The reaction mixture was stirred at room temperature under hydrogen (balloon) atmosphere and monitored by thin layer chromatography. Upon completion of the starting material, the reaction mixture was filtered through a pad of silica gel and eluted with ethyl acetate (20 mL × 2). The combined organic filtrate was washed with brine (30 mL), dried over anhydrous sodium sulfate and concentrated in vacuo to afford compound **16** (3.46 g, 97%) as an oil. An analytical sample was obtained by chromatography. [α]${}_{D}^{20}$ = 1.5 (*c* 1.1, CHCl_3_); ^1^H-NMR (500 MHz, CDCl_3_) δ 7.48–7.35 (m, 5H), 7.16 (d, *J* = 8.0 Hz, 2H), 6.95 (d, *J* = 8.0 Hz, 2H), 5.07 (s, 2H), 4.19–4.08 (m, 1H), 4.04 (dd, *J* = 7.9, 5.8 Hz, 1H), 3.56 (t, *J* = 7.5 Hz, 1H), 2.79–2.60 (m, 2H), 2.04–1.73 (m, 2H), 1.49 (s, 3H), 1.41 (s, 3H). ^13^C-NMR (125 MHz, CDCl_3_) δ 157.16, 137.21, 133.92, 129.34, 128.61, 127.95, 127.51, 114.84, 108.74, 75.41, 70.05, 69.40, 35.59, 31.19, 27.08, 25.83 ppm; HRMS (ESI) calculated for C_20_H_24_O_3_ [M + Na]^+^ 335.1725, found 335.1744.

Compound **16** (0.50 g, 1.60 mmol) was dissolved in methanol (10 mL) and cooled to 0 °C, after PTSA (30 mg, 0.16 mmol) was added, the reaction mixture was stirred at room temperature for 16 h. The reaction solution was concentrated in vacuo, the residue was dissolved in ethyl acetate (50 mL) and washed with saturated aqueous solution of sodium bicarbonate (50 mL) and brine (50 mL). The organic layer was dried over anhydrous sodium sulfate and concentrated in vacuo. The residue was purified by flash chromatography to afford the desired diol compound **S1** (0.43 g, 99%) as clear oil. [α]${}_{D}^{20}$ = −14.7 (*c* 1.0, MeOH); ^1^H-NMR (500 MHz, CDCl_3_) δ 7.47–7.29 (m, 5H), 7.12 (d, *J* = 8.5 Hz, 2H), 6.91 (d, *J* = 8.6 Hz, 2H), 5.04 (s, 2H), 3.78–3.57 (m, 2H), 3.46 (dd, *J* = 11.1, 7.6 Hz, 1H), 2.80–2.55 (m, 2H), 2.28 (s, 1H), 2.08 (s, 1H), 1.72 (m, 2H). ^13^C NMR (125 MHz, CDCl_3_) δ 157.16, 137.20, 134.01, 129.33, 129.31, 128.57, 128.55, 127.89, 127.47, 127.44, 114.92, 114.88, 77.27, 77.01, 76.76, 71.52, 70.12, 66.82, 34.87, 30.90 ppm; HRMS (ESI) calculated for C_17_H_20_O_3_ [M + Na]^+^ 295.1412, found 295.1456.

Diol compound **S1** (136 mg, 0.50 mmol) was dissolved in DCM (5 mL) and cooled to 0 °C, after triethyl amine (0.35 mL, 2.5 mmol) and triphenylmethyl chloride (0.15 g, 0.53 mmol) were added, the reaction mixture was stirred at room temperature for 4 h. The reaction was diluted with DCM (50 mL) and extracted with brine (50 mL). Layers were separated, the organic phase was dried over anhydrous sodium sulfate, concentrated in vacuo, and purified by flash chromatography to afford compound **17** (0.24 g, 93%) as clear oil. [α]${}_{D}^{20}$ = 2.7 (*c* 1.0, CHCl_3_); ^1^H-NMR (500 MHz, CDCl_3_) δ 7.52–7.21 (m, 20H), 7.09 (d, *J* = 8.5 Hz, 2H), 6.91 (d, *J* = 8.5 Hz, 2H), 5.06 (s, 2H), 3.81 (s, 1H), 3.21 (dd, *J* = 9.4, 3.3 Hz, 1H), 3.09 (dd, *J* = 9.4, 7.3 Hz, 1H), 2.74–2.52 (m, 2H), 2.36 (s, 1H), 1.83–1.64 (m, 2H). ^13^C-NMR (125 MHz, CDCl_3_) δ 157.08, 143.90, 137.30, 134.31, 129.37, 128.72, 128.59, 127.90, 127.48, 127.15, 114.83, 86.73, 70.23, 70.12, 67.71, 35.20, 30.83 ppm; HRMS (ESI) calculated for C_36_H_34_O_3_ [M + Na]^+^ 537.2508, found 537.2553.

To a solution of compound **17** (0.43 g, 0.84 mmol) dissolved in THF (3 mL), NaH (0.1g, 4.17 mmol) was added at 0 °C. The reaction mixture was stirred at 0 °C for 0.5 h, then benzyl bromide (0.15 mL, 1.25 mmol) was added. Upon completion of the addition, the reaction mixture was brought to room temperature and stirred for 16 h. The reaction was quenched by saturated aqueous solution of NH_4_Cl (5 mL). Volatiles were removed in vacuo. The residue was extracted with ethyl acetate (50 mL × 2). The combined organic phases were washed with brine (50 mL), dried over anhydrous sodium sulfate and concentrated in vacuo. The residue was purified by flash chromatography to give the desired fully protected compound **S2** (0.48 g, 95%) as clear oil. [α]${}_{D}^{20}$ = −2.3 (*c* 1.0, CHCl_3_); ^1^H-NMR (400 MHz, CDCl_3_) δ 7.56–7.17 (m, 25H), 7.09 (d, *J* = 8.5 Hz, 2H), 6.91 (d, *J* = 8.5 Hz, 2H), 5.04 (s, 2H), 4.71 (d, *J* = 11.6 Hz, 1H), 4.50 (d, *J* = 11.6 Hz, 1H), 3.54 (dt, *J* = 9.2, 4.7 Hz, 1H), 3.21 (qd, *J* = 9.9, 4.8 Hz, 2H), 2.67–2.48 (m, 2H), 1.93–1.80 (m, 2H). ^13^C-NMR (100 MHz, CDCl_3_) δ 156.95, 144.13, 138.88, 137.24, 134.58, 129.32, 128.75, 128.56, 128.35, 127.88, 127.77, 127.47, 126.94, 114.71, 86.61, 77.73, 72.04, 70.06, 65.79, 34.02, 30.71 ppm; HRMS (ESI) calculated for C_43_H_40_O_3_ [M + Na]^+^ 627.2977, found 627.2941.

The above intermediate **S2** (0.50 g, 0.83 mmol) was dissolved in methanol–DCM (4 mL, 1:1) and cooled to 0 °C. After PTSA (16 mg, 0.08 mmol) was added, the reaction mixture was stirred at room temperature for 4 h. Then the reaction was concentrated in vacuo. The residue was purified by flash chromatography to produce compound **18** (0.21 g, 71%). [α]${}_{D}^{20}$ = 2.0 (*c* 1.1, CHCl_3_); ^1^H-NMR (400 MHz, CDCl_3_) δ 7.49–7.29 (m, 10H), 7.09 (d, *J* = 8.5 Hz, 2H), 6.91 (d, *J* = 8.5 Hz, 2H), 5.06 (s, 2H), 4.66–4.53 (m, 2H), 3.75 (dd, *J* = 11.2, 3.1 Hz, 1H), 3.64–3.48 (m, 2H), 2.66 (ddd, *J* = 8.8, 6.6, 4.7 Hz, 2H), 2.03–1.76 (m, 2H). ^13^C-NMR (100 MHz, CDCl_3_) δ 157.08, 138.38, 137.18, 134.17, 129.27, 128.57, 128.53, 127.92, 127.86, 127.83, 127.48, 114.85, 78.96, 71.58, 70.08, 64.13, 32.81, 30.70 ppm; HRMS (ESI) calculated for C_24_H_26_O_3_ [M + Na]^+^ 385.1882, found 385.1920.

Compound **18** (0.21 g, 0.59 mmol) was dissolved in DCM–H_2_O (3 mL, 2:1) at 0 °C. DAIB (16 mg, 0.08 mmol) and TEMPO (5 mg, 0.29 mmol) were sequentially added, the reaction mixture was stirred in dark for 16 h at room temperature. The reaction was diluted by DCM (50 mL) and washed with brine (20 mL). The organic layer was separated, dried over sodium sulfate, and concentrated in vacuo. The residue was purified by flash chromatography to provide acid ***S*-2** (0.19 g, 84%) as clear oil. [α]${}_{D}^{20}$ = −15.5 (*c* 1.0, CHCl_3_); ^1^H-NMR (500 MHz, CDCl_3_) δ 7.47–7.30 (m, 10H), 7.09 (d, *J* = 8.5 Hz, 2H), 6.91 (d, *J* = 8.5 Hz, 2H), 5.04 (s, 2H), 4.74 (d, *J* = 11.5 Hz, 1H), 4.45 (d, *J* = 11.4 Hz, 1H), 3.98 (t, *J* = 6.2 Hz, 1H), 2.72 (ddt, *J* = 43.4, 13.9, 7.7 Hz, 2H), 2.14–2.07 (m, 2H). ^13^C-NMR (125 MHz, CDCl_3_) δ 177.06, 157.24, 137.23, 133.32, 129.46, 128.56, 128.53, 128.16, 128.07, 127.89, 127.46, 114.93, 72.58, 70.12, 34.49, 30.42 ppm; HRMS (ESI) calculated for C_24_H_24_O_4_ [M + H]^+^ 377.1675, found 377.1695.

#### 4.3.2. Synthesis of *r*-Hhpba (**21**)

Compound ***S*-2** (40 mg, 0.11 mmol) was dissolved in DMF (2 mL) at 0 °C, Cs_2_CO_3_ (30 mg, 0.21 mmol) and CH_3_I (33 μL, 0.53 mmol) were added at 0 °C. The reaction was brought to room temperature and stirred for 16 h. The reactant was diluted by ethyl acetate (50 mL). The organic solution was washed with brine (30 mL × 3), dried over anhydrous sodium sulfate, and concentrated in vacuo. The residue was purified by flash chromatography to give the corresponding methyl ester **S3** (40 mg, 96%) as an oil. [α]${}_{D}^{20}$ = −19.2 (*c* 1.1, CHCl_3_); ^1^H-NMR (500 MHz, CDCl_3_) δ 7.47–7.31 (m, 10H), 7.09 (d, *J* = 8.5 Hz, 2H), 6.91 (d, *J* = 8.5 Hz, 2H), 5.05 (s, 2H), 4.74 (d, *J* = 11.5 Hz, 1H), 4.42 (d, *J* = 11.5 Hz, 1H), 3.95 (dd, *J* = 8.2, 4.7 Hz, 1H), 3.74 (s, 3H), 2.81–2.63 (m, 2H), 2.12–2.02 (m, 2H). ^13^C-NMR (125 MHz, CDCl_3_) 173.28, 157.22, 137.59, 137.27, 133.44, 129.48, 128.57, 128.45, 128.15, 128.13, 127.90, 127.46, 114.91, 72.44, 70.12, 70.09, 51.84, 34.75, 30.52 ppm; HRMS (ESI) calculated for C_25_H_26_O_4_ [M + H]^+^ 391.1831, found 391.1889.

Methyl ester **S3** (60 mg, 0.154 mmol) was dissolved in ethyl acetate (2 mL), after Pd/C (6 mg, 10% Pd on charcoal) was added under a protective flow of nitrogen, the reaction vessel was sealed and purged by hydrogen. The reaction mixture was stirred under hydrogen atmosphere (balloon) for 1 h. The reaction was monitored by TLC. Upon the complete consumption of starting material, the reaction solution was filtrated through a pad of celite and rinsed by ethyl acetate (5 mL). The combined filtrate was concentrated in vacuo to produce the desired compound **S4** as colorless oil. An analytical sample was obtained via chromatography. [α]${}_{D}^{20}$ = 13.9 (*c* 1.0, CHCl_3_); ^1^H-NMR (300 MHz, CDCl_3_) δ 7.09 (d, *J* = 8.5 Hz, 2H), 6.91 (d, *J* = 8.5 Hz, 2H), 5.80 (s, 1H), 4.21 (dd, *J* = 8.2, 4.1 Hz, 1H), 3.76 (s, 3H), 3.09 (d, *J* = 5.5 Hz, 1H), 2.75–2.58 (m, 2H), 2.25–1.83 (m, 2H). ^13^C-NMR (75 MHz, CDCl_3_) δ 175.66, 153.88, 132.74, 129.56, 115.21, 69.60, 52.56, 35.93, 29.95 ppm; HRMS (ESI) calculated for C_11_H_14_O_4_ [M + Na]^+^ 233.0892, found 233.0823.

Intermediate **S4**, obtained from last step of reaction without further purification, was dissolved in acetonitrile (2 mL) at 0 °C. K_2_CO_3_ (30 mg, 0.21 mmol) was added to the solution, 10 min later, benzyl bromide (85 μL, 0.72 mmol) and TBAI (5 mg, 0.014 mmol) were sequentially added. The reaction mixture was stirred at room temperature for 16 h, and quenched by addition of saturated aqueous solution of NH_4_Cl (10 mL). Volatiles were removed in vacuo. The residue was extracted by ethyl acetate (30 mL × 2). The combined organic layers were washed with brine (20 mL), dried over anhydrous sodium sulfate, and concentrated in vacuo. The residue was purified by flash chromatography to afford compound **19** (40 mg, 88%). [α]${}_{D}^{20}$ = 10.9 (*c* 1.0, CHCl_3_); ^1^H-NMR (300 MHz, CDCl_3_) δ 7.54–7.30 (m, 5H), 7.09 (d, *J* = 8.5 Hz, 2H), 6.91 (d, *J* = 8.5 Hz, 2H), 5.06 (s, 2H), 4.20 (dd, *J* = 8.0, 4.1 Hz, 1H), 3.76 (s, 3H), 2.91–2.60 (m, 3H), 2.22–1.80 (m, 2H). ^13^C-NMR (75 MHz, CDCl_3_) δ 175.61, 157.06, 137.06, 133.26, 129.43, 128.47, 127.82, 127.37, 114.73, 69.95, 69.49, 52.46, 35.97, 29.99 ppm; HRMS (ESI) calculated for C_18_H_20_O_4_ [M + Na]^+^ 323.1362, found 323.1302.

Triphenylphosphine (40 mg, 0.133 mmol) was added to a solution of compound **19** (10 mg, 0.033 mmol) and 4-nitrobenzoic acid (11 mg, 0.066 mmol) dissolved in THF (2 mL) at 0 °C, after DEAD (10 μL, 0.066 mmol) was added, the reaction was stirred at room temperature for 16 h and then quenched by addition of saturated aqueous solution of NH_4_Cl (10 mL). Volatiles were removed in vacuo. The residue was extracted by ethyl acetate (30 mL × 2). The combined organic layers were washed with brine (20 mL), dried over anhydrous sodium sulfate, and concentrated in vacuo. The residue was purified by flash chromatography to produce ester **20** (12 mg, 78%) as an oil. ^1^H-NMR (300 MHz, CDCl_3_) δ 8.39–8.12 (m, 4H), 7.53–7.30 (m, 5H), 7.09 (d, *J* = 8.5 Hz, 2H), 6.91 (d, *J* = 8.5 Hz, 2H), 5.29 (t, *J* = 6.3 Hz, 1H), 5.05 (s, 2H), 3.78 (s, 3H), 2.80 (t, *J* = 7.6 Hz, 2H), 2.41–2.24 (m, 2H). ^13^C-NMR (75 MHz, CDCl_3_) δ 169.98, 164.04, 157.32, 150.69, 136.89, 134.61, 132.25, 130.92, 129.30, 128.51, 127.90, 127.39, 123.50, 114.90, 72.80, 69.97, 52.47, 32.66, 30.54 ppm; HRMS (ESI) calculated for C_25_H_23_NO_7_ [M + Na]^+^ 472.1475, found 472.1515.

K_2_CO_3_ (6 mg, 0.044 mmol) was added to a solution of compound **20** (33 mg, 0.073 mmol) dissolved in methanol (2 mL) at 0 °C, the reaction mixture was then brought to room temperature and stirred for 10 h. Volatiles were removed in vacuo, the residue was dissolved in ethyl acetate (50 mL). The organic solution was washed with brine (30 mL), dried over anhydrous sodium sulfate and concentrated in vacuo. The residue was purified by flash chromatography to afford the secondary alcohol compound ***ent***-**19** (20 mg, 85%). [α]${}_{D}^{20}$ = −10.2 (*c* 1.0, CHCl_3_).

LiOH (7 mg, 0.17 mmol) was added to a solution of the above secondary alcohol (10 mg, 33 μmol) dissolved in THF-MeOH-H_2_O (3 mL, *v*/*v*/*v* = 1/1/1) at 0 °C. The reaction mixture was stirred at room temperature for 3 h and concentrated in vacuo. The residue was diluted with water (5 mL) and extracted with Et_2_O (20 mL), the organic phase was discarded, the aqueous phase was adjusted to pH 3 with dilute HCl and extracted by ethyl acetate (30 mL × 3). The combined organic layers were washed with brine (30 mL), dried over anhydrous sodium sulfate, and concentrated in vacuo to give acid **21** (8 mg, 91%), which was ready for next step of transformation.

### 4.4. Synthesis of Nostosin B ***1a*** and ***1b***

***S*-2** (20 mg, 0.053 mmol) and **7** (51 mg, 0.08 mmol) were dissolved in DCM (2 mL) at 0 °C, HOAT (14 mg, 0.11 mmol) and HATU (40 mg, 0.11 mmol) were added, followed by addition of DIPEA (44 μL, 0.26 mmol). The reaction mixture was warmed to room temperature and stirred for 16 h. Volatiles were removed in vacuo. The residue was dissolved in ethyl acetate (100 mL), washed with saturated aqueous solution of NH_4_Cl (30 mL), NaHCO_3_ (30 mL) and brine (30 mL), dried over Na_2_SO_4_, and concentrated in vacuo. The residue was purified by flash chromatography (EA/Hexanes) to provide tripeptide **22** (45 mg, 85%) as a viscous oil. [α]${}_{D}^{20}$ = −25.5 (*c* 1.0, CHCl_3_); ^1^H-NMR (500 MHz, CDCl_3_) δ 7.51–7.27 (m, 10H), 7.20 (d, *J* = 8.5 Hz, 1H), 7.09 (d, *J* = 8.5 Hz, 2H), 6.91 (d, *J* = 8.5 Hz, 2H), 6.41 (d, *J* = 8.7 Hz, 1H), 6.09 (br, 2H), 5.03 (s, 2H), 4.60 (d, *J* = 11.1 Hz, 1H), 4.45 (d, *J* = 11.1 Hz, 1H), 4.21 (t, *J* = 7.8 Hz, 1H), 3.95 (brs, 1H), 3.86 (dd, *J* = 7.2, 4.2 Hz, 1H), 3.69–3.46 (m, 2H), 3.28–3.05 (m, 2H), 2.92 (s, 2H), 2.68–2.60 (m, 2H), 2.57 (s, 3H), 2.51 (s, 3H), 2.07 (s, 3H), 2.05–1.92 (m, 2H), 1.86–1.72 (m, 1H), 1.57–1.43 (m, 4H), 1.44 (s, 6H), 1.18–1.06 (m, 1H), 0.92 (d, *J* = 6.7 Hz, 3H), 0.88 (brs, 12H), 0.04 (s, 3H); 0.03 (s, 3H). ^13^C-NMR (125 MHz, CDCl_3_) δ 173.29, 171.24, 158.58, 157.25, 156.12, 138.34, 137.22, 136.96, 133.46, 132.31, 129.34, 128.68, 128.53, 128.28, 128.17, 127.86, 127.47, 127.43, 124.43, 117.31, 114.95, 86.21, 79.42, 72.80, 70.11, 65.10, 57.87, 50.36, 43.29, 41.09, 36.98, 34.83, 30.22, 29.24, 28.57, 25.86, 25.17, 25.11, 19.20, 18.28, 17.85, 15.53, 12.40, 11.08, −5.49, −5.52 ppm; HRMS (ESI) calculated for C_55_H_79_N_5_O_8_SSi [M + H]^+^ 998.5419, found 998.5486.

Tripeptide **22** (72 mg, 0.072 mmol) was dissolved in ethyl acetate (2 mL), Pd/C (8 mg, 10%) was added. Hydrogen was bubbled for 1 h at room temperature. The conversion of reaction was monitored by TLC. The mixture was concentrated and passed through a silica column. The filtrate was concentrated in vacuo, the residue was dried under high vacuum for 2 h and then it (56 mg, 0.068 mmol) was dissolved in DCM (2 mL), TFA (0.5 mL) was added at 0 °C. The reaction was stirred at room temperature for 2 h, volatiles were removed in vacuo. The residue was purified by HPLC and lyophilized to give nostosin B **1a** (17 mg, 74%) as a powder. (Welch Materials. Inc. XB-C18, 5 μm, 4.6 × 250 mm, 1.0 mL/min, 18% MeCN:82% H_2_O within 30 min, DAD detector was adjusted to 195 nm wave length.). [α]${}_{D}^{20}$ = 1.6 (*c* 1.0, MeOH); ^1^H-NMR (400 MHz, DMSO-*d*_6_) δ 9.14 (brs, 1H), 7.86 (d, *J* = 8.4 Hz, 1H), 7.50–7.46 (m, 2H), 6.94 (d, *J* = 8.4 Hz, 2H), 6.64 (d, *J* = 8.4 Hz, 2H), 5.74 (brs, 1H), 4.65 (brs, 1H), 4.18 (dd, *J* = 9.2, 6.9 Hz, 1H), 3.84 (dd, *J* = 7.9, 3.8 Hz, 1H), 3.69 (td, *J* = 8.6, 3.8 Hz, 1H), 3.12–2.99 (m, 2H), 2.52–2.49 (m, 2H), 1.86–1.82 (m, 1H), 1.74–1.63 (m, 1H), 1.59–1.54 (m, 1H), 1.48–1.32 (m, 3H), 1.33–1.22 (m, 2H), 1.08–0.95 (m, 2H), 0.84–0.78 (m, 6H). ^13^C-NMR (100 MHz, DMSO-*d_6_*) δ 173.69, 171.06, 157.07, 155.78, 132.21, 129.56, 115.53, 70.86, 63.61, 56.41, 50.81, 41.17, 37.95, 37.39, 30.32, 28.24, 25.67, 24.67, 15.80, 11.46 ppm; HRMS (ESI) calculated for C_22_H_37_N_5_O_5_ [M + H]^+^ 452.2795, found 452.2856.

Compound **21** (8 mg, 0.03 mmol) and **7** (28 mg, 0.045 mmol) were dissolved in DCM (2 mL) at 0 °C, after HATU (23 mg, 0.06 mmol), HOAt (8 mg, 0.06 mmol) were added followed by addition of DIPEA (25 μL, 0.15 mmol), the reaction mixture was stirred at room temperature for 16 h. The reaction was concentrated in vacuo, the residue was dissolved in DCM (75 mL), the organic solution was washed sequentially by saturated aqueous solution of NH_4_Cl (30 mL), NaHCO_3_ (30 mL) and brine (30 mL). The organic layer was separated, dried over anhydrous sodium sulfate, and concentrated in vacuo. The residue was purified by flash chromatography to produce compound **23** (23 mg, 86%). [α]${}_{D}^{20}$ = −13.8 (*c* 1.0, CHCl_3_); ^1^H-NMR (500 MHz, CHCl_3_) δ 7.45–7.28 (m, 6H), 7.09 (d, *J* = 8.0 Hz, 2H), 6.91–6.82 (d, *J* = 8.0 Hz, 2H), 6.50 (d, *J* = 9.3 Hz, 1H), 6.36–6.21 (m, 2H), 6.00 (brs, 1H), 5.00 (s, 2H), 4.19 (t, *J* = 8.4 Hz, 1H), 4.10 (dd, *J* = 8.4, 3.4 Hz, 1H), 3.96 (brs, 1H), 3.54 (dd, *J* = 8.4, 4.2 Hz, 2H), 3.25–3.00 (m, 2H), 2.92 (s, 2H), 2.76–2.61 (m, 2H), 2.55 (s, 3H), 2.49 (s, 3H), 2.07 (s, 3H), 1.93–1.81 (m, 3H), 1.62–1.37 (m, 11H), 1.17–1.12 (m, 1H), 0.92–0.86 (m, 15H), 0.03 (s, 6H). ^13^C-NMR (125 MHz, CDCl_3_) δ 174.58, 171.80, 158.77, 157.17, 156.23, 138.40, 137.21, 133.50, 132.87, 132.33, 129.52, 128.53, 127.87, 127.47, 124.60, 117.48, 114.89, 86.36, 71.33, 70.05, 65.17, 58.09, 43.23, 40.90, 36.66, 36.40, 30.43, 29.69, 28.82, 28.58, 25.87, 25.29, 25.09, 19.23, 18.30, 17.88, 15.42, 12.45, 11.00, −5.47, −5.51 ppm; HRMS (ESI) calculated for C_48_H_73_N_5_O_8_SSi [M + H]^+^ 908.4949, found 908.5102.

Compound **23** (16 mg, 0.0176 mmol) was dissolved in ethyl acetate (2 mL), after Pd/C (5 mg, 10% Pd on charcoal) was added under a protective nitrogen flow, the reaction vessel was sealed and flashed by hydrogen. The reaction mixture was stirred under hydrogen atmosphere (balloon) at room temperature for 1 h, then it was filtered through a pad of celite to remove the catalyst, the filter cake was rinsed by ethyl acetate (5 mL × 2). The combined filtrate was concentrated in vacuo. The residue was dried under high vacuum for 2 h, and it (13 mg, 0.016 mmol) was subsequently dissolved in DCM (2 mL) at 0 °C, after TFA (0.5 mL) was added, the reaction mixture was stirred at room temperature for 2 h. Volatiles were removed in vacuo, the residue was purified by HPLC and lyophilized to give nostosin B **1b** (6 mg, 75%) as a powder. (Agilent 1200; Column: Welch materials (Shanghai) Inc., Shanghai, China, XB-C18, 5 μm, 4.6 × 250 mm; flow rate: 1.0 mL/min; eluent: 18% CH_3_CN with 82% H_2_O; detector: DAD at 195 nm^−1^) [α]${}_{D}^{20}$ = −2.4 (*c* 1.1, MeOH); ^1^H-NMR (500 MHz, DMSO-*d_6_*) δ 9.13 (s, 1H), 7.84 (d, *J* = 8.5 Hz, 1H), 7.59 (d, *J* = 9.0 Hz, 1H), 7.54 (t, *J* = 6.1 Hz, 1H), 6.94 (d, *J* = 8.3 Hz, 2H), 6.65 (d, *J* = 8.3 Hz, 2H), 5.69 (d, *J* = 5.6 Hz, 1H), 4.70 (t, *J* = 5.3 Hz, 1H), 4.16–4.12 (m, 1H), 3.88 (ddd, *J* = 8.9, 5.7, 3.8 Hz, 1H), 3.69 (dq, *J* = 9.2, 4.1 Hz, 1H), 3,37-3.30 (m, 1H), 3.23–3.19 (m, 1H), 3.03 (dt, *J* = 12.1, 6.7 Hz, 2H), 2.52–2.46 (m, 2H), 1.87–1.78 (m, 1H), 1.72–1.54 (m, 3H), 1.52–1.36 (m, 3H), 1.33–1.24 (m, 1H), 1.08–1.00 (m, 1H), 0.85–0.75 (m, 6H). ^13^C-NMR (125 MHz, DMSO-*d_6_*) δ 173.41, 170.69, 156.65, 155.27, 131.75, 129.07, 115.03, 70.10, 63.17, 56.42, 50.23, 40.68, 37.02, 36.52, 29.86, 27.79, 25.10, 24.36, 15.28, 10.91 ppm; HRMS (ESI) calculated for C_22_H_37_N_5_O_5_ [M + H]^+^ 452.2795, found 452.2885.

## 5. Conclusions

We have successfully completed the total synthesis of nostosin B and unambiguously defined the absolute stereochemistry of 2-hydroxy-4-(4-hydroxyphenyl)butanoic acid (Hhpba) fragment as *r*-configuration. Both NMR spectra data and optical rotation value for norstosin B **1b** matched the natural product. Additional proofs came from the direct comparison of **1b** with the authentic sample using HPLC techniques. Synthetic nostosin B **1b** showed identical retention time with the natural product on both reversed phase column and the chiral AD-RH column. Further evaluations of the biological activity of nostosins are currently being undertaken in this laboratory and will be reported in due course.

## Figures and Tables

**Table 1:** Screening of the reaction conditions.

Entry	Lewis Acid	Solvent	Temperature (°C)	Results
1	AlCl_3_	Toluene	reflux	decomposed
2	AlCl_3_	Toluene	room temperature	decomposed
3	AlCl_3_	DCE	reflux	decomposed
4	AlCl_3_	DCE	room temperature	decomposed
5	BF_3_·Et_2_O	DCE	room temperature	decomposed
6	4Å MS	DCE	room temperature	no reaction

## Supplementary Materials

The NMR Spectra of key intermediates are available online at www.mdpi.com/1660-3397/15/3/58/s1.
