# Supplementary material for: Total Synthesis and Stereochemical Assignment of Nostosin B
Source: Mar Drugs. 2017 Feb 27;15(3):58. doi: 10.3390/md15030058 (PMC5367015; doi:10.3390/md15030058)

Supplementary Spectra

Total Synthesis and Stereochemical Assignment of Nostosin B

NMR spectra of compound 6 (Bruker 500 MHz, $CDCl_3$)

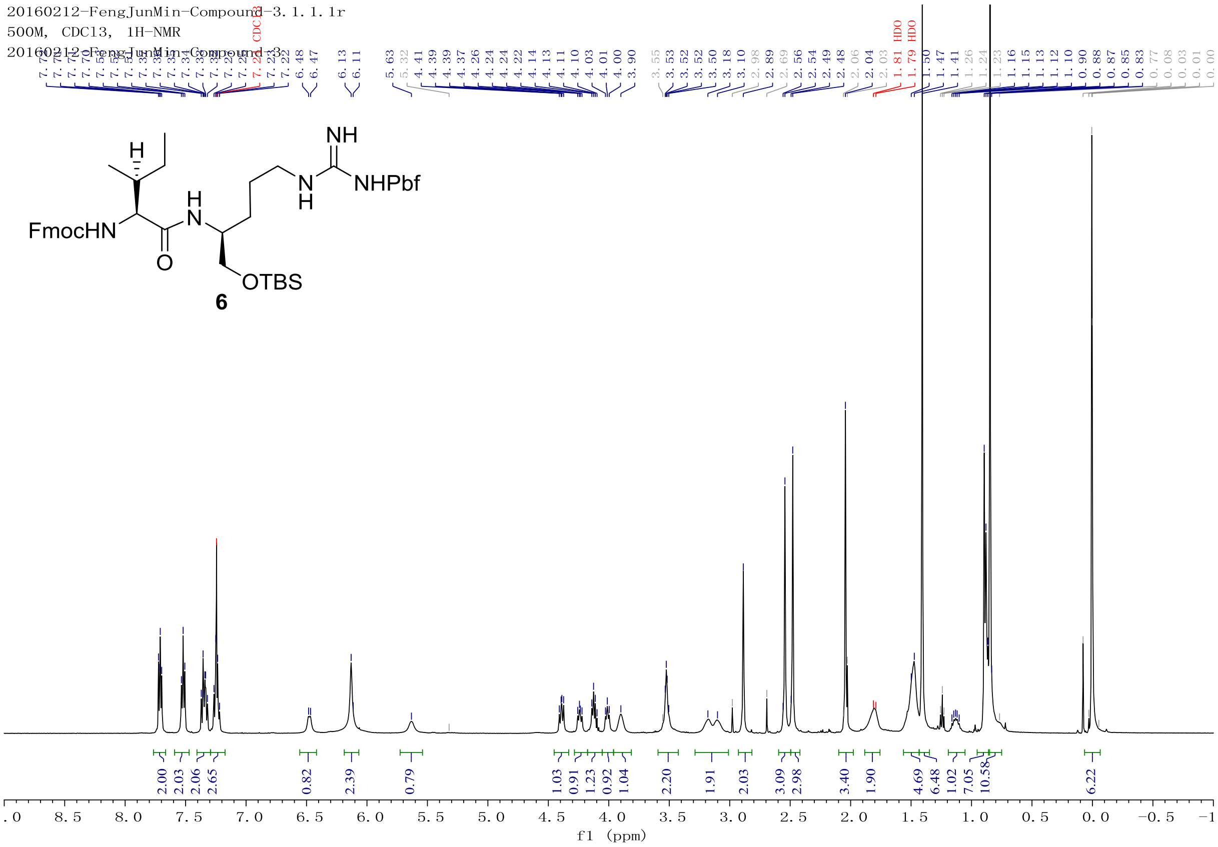

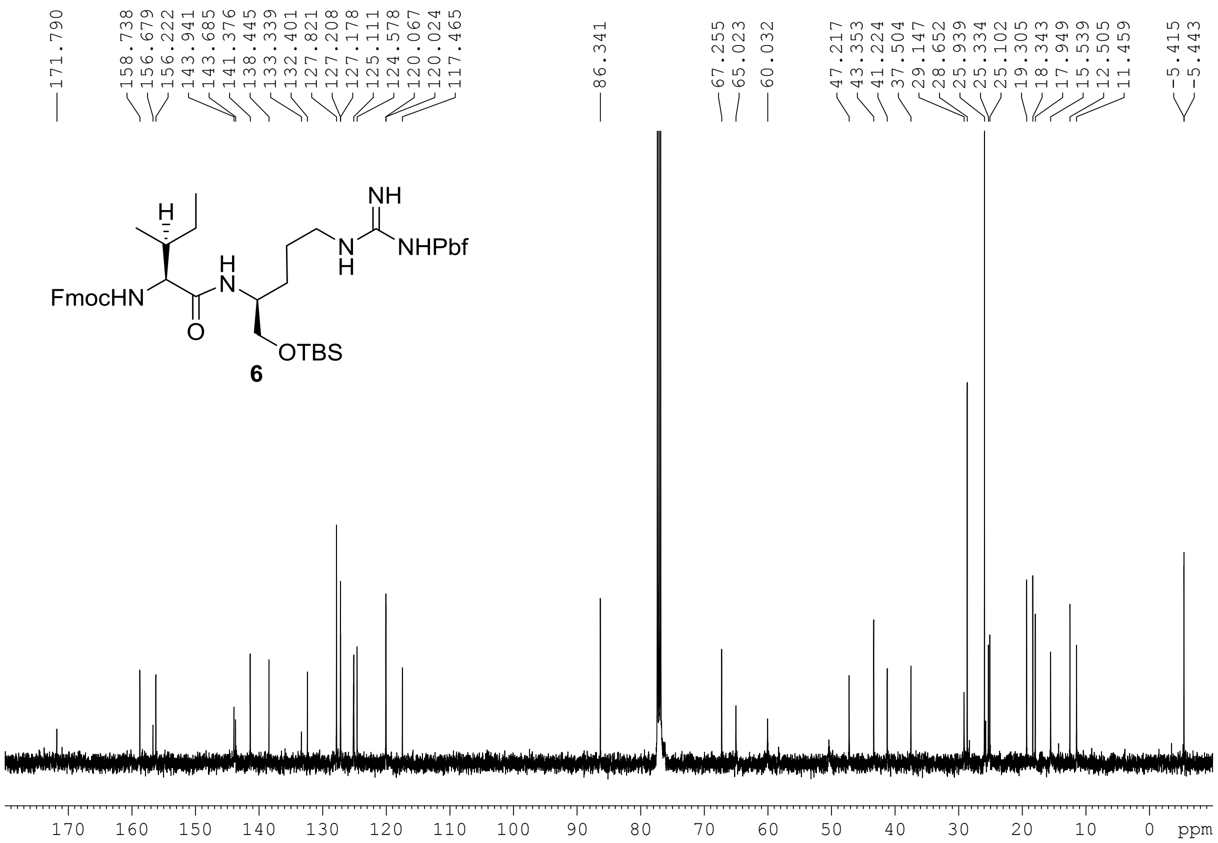

NMR spectra of compound 16 (Bruker 500 MHz, $CDCl_3$)

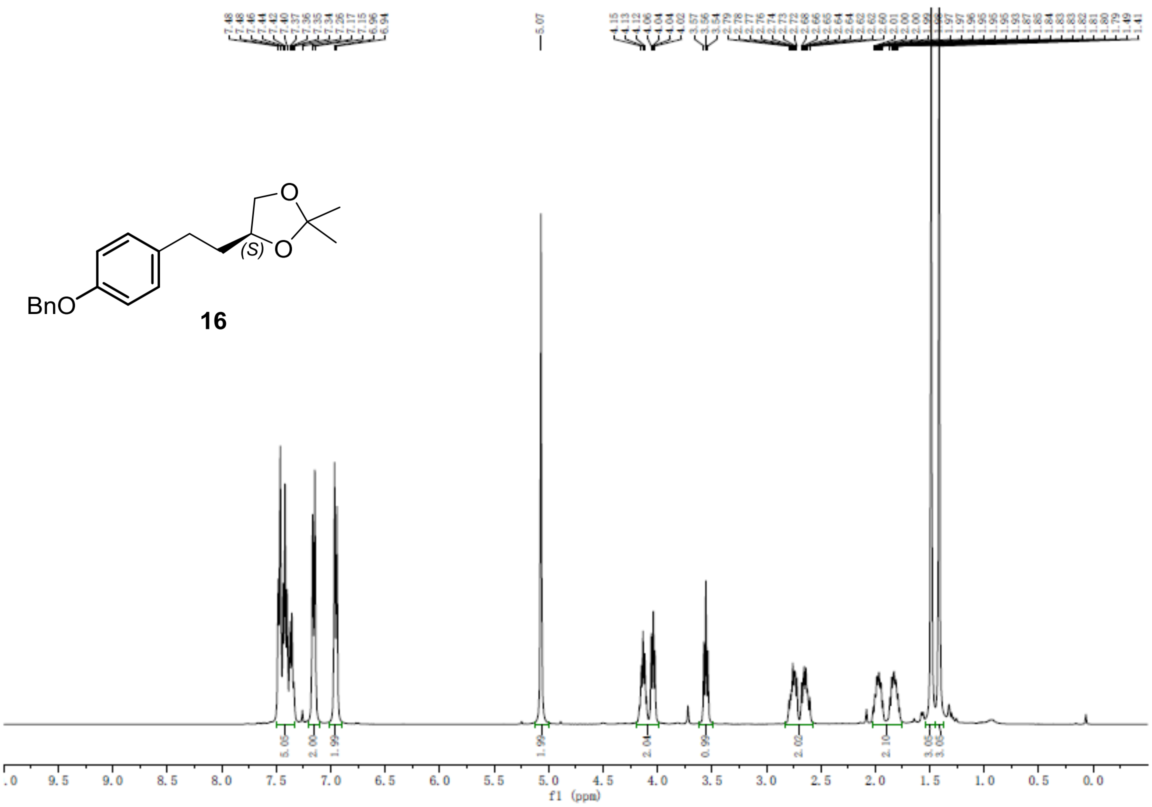

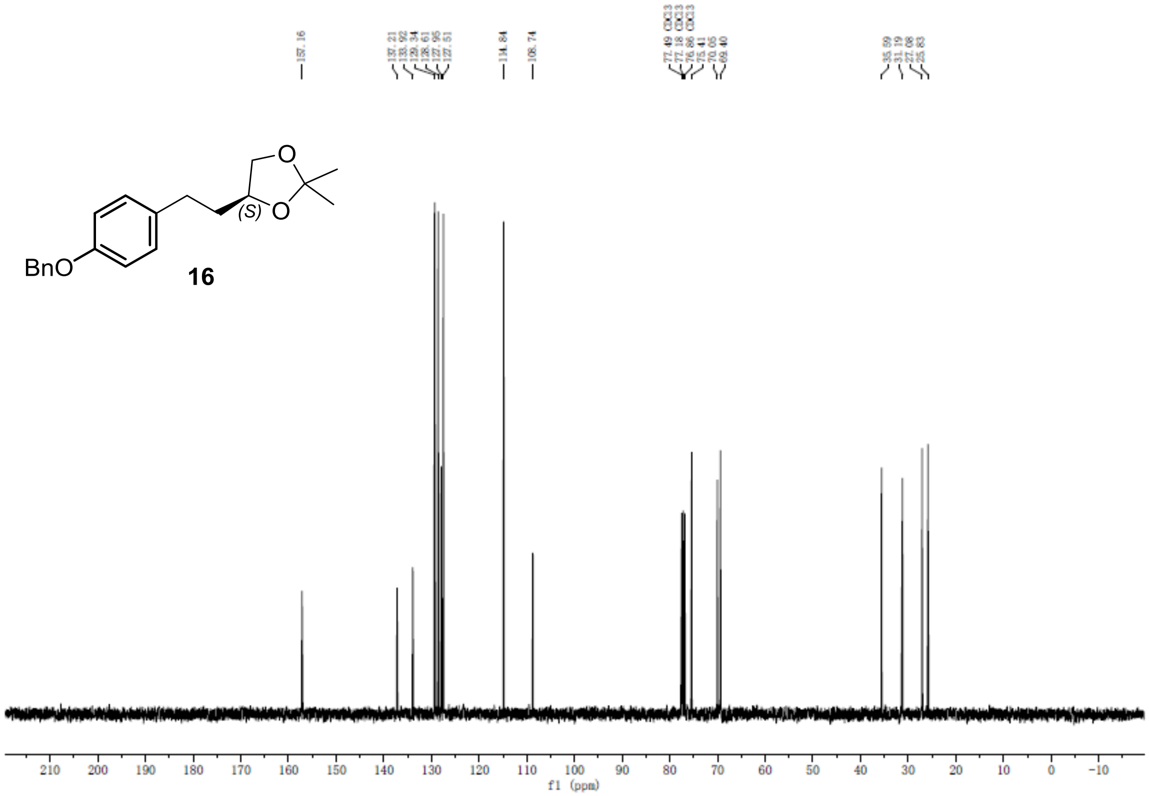

NMR spectra of compound S1 (Bruker 500 MHz, $CDCl_3$)

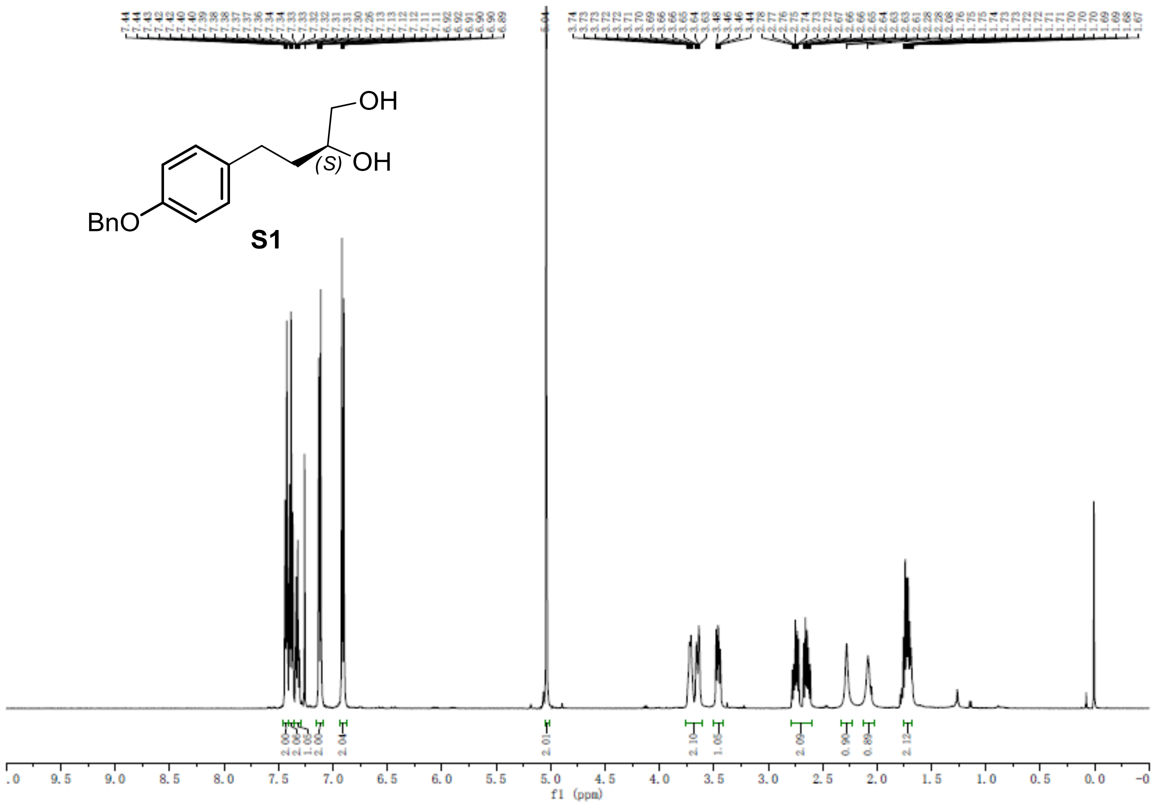

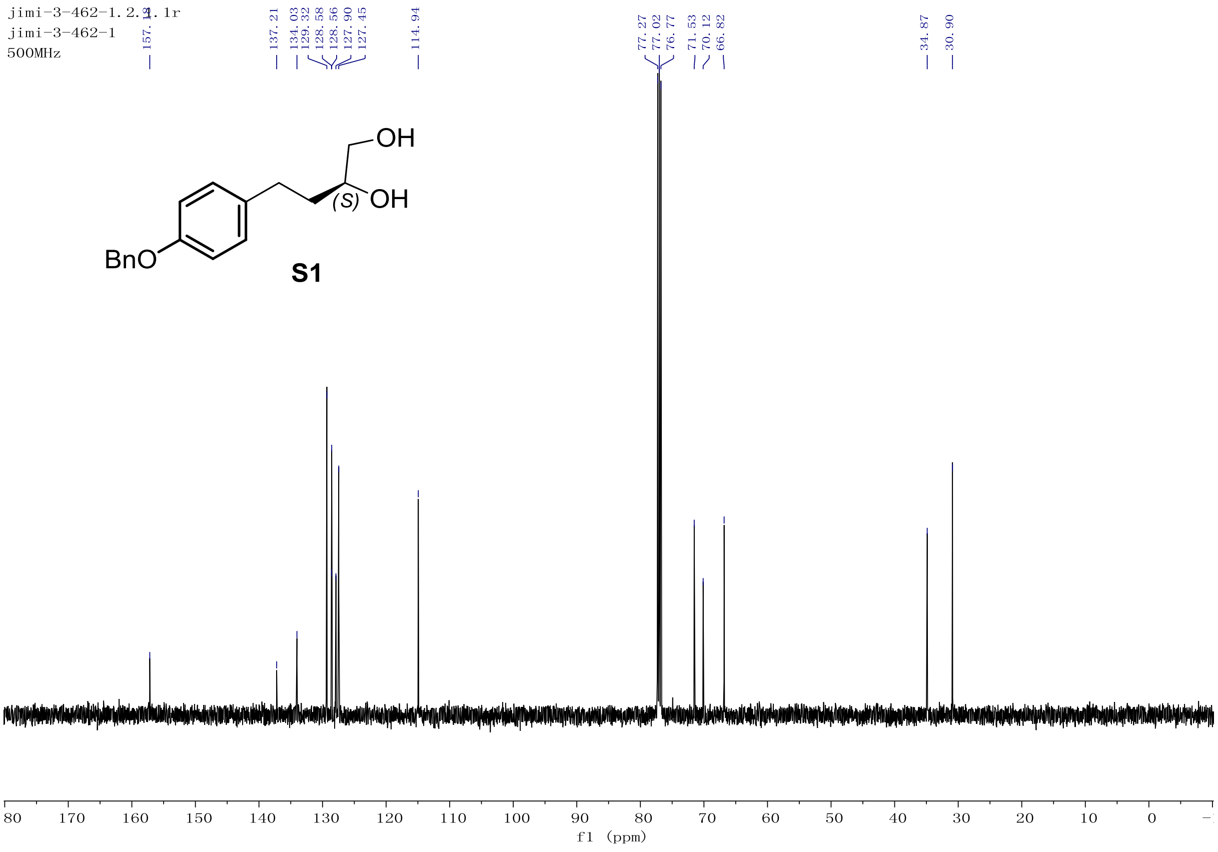

NMR spectra of compound 17 (Bruker 500 MHz, $CDCl_3$)

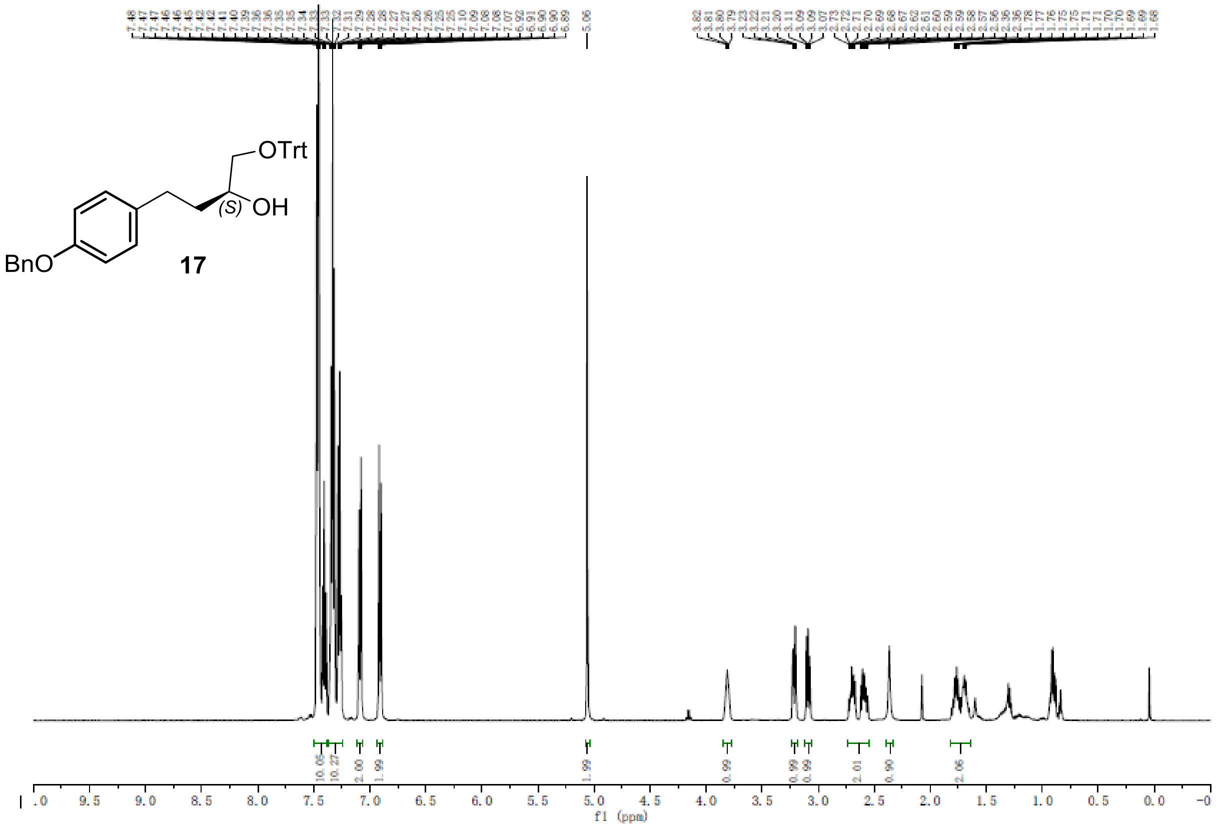

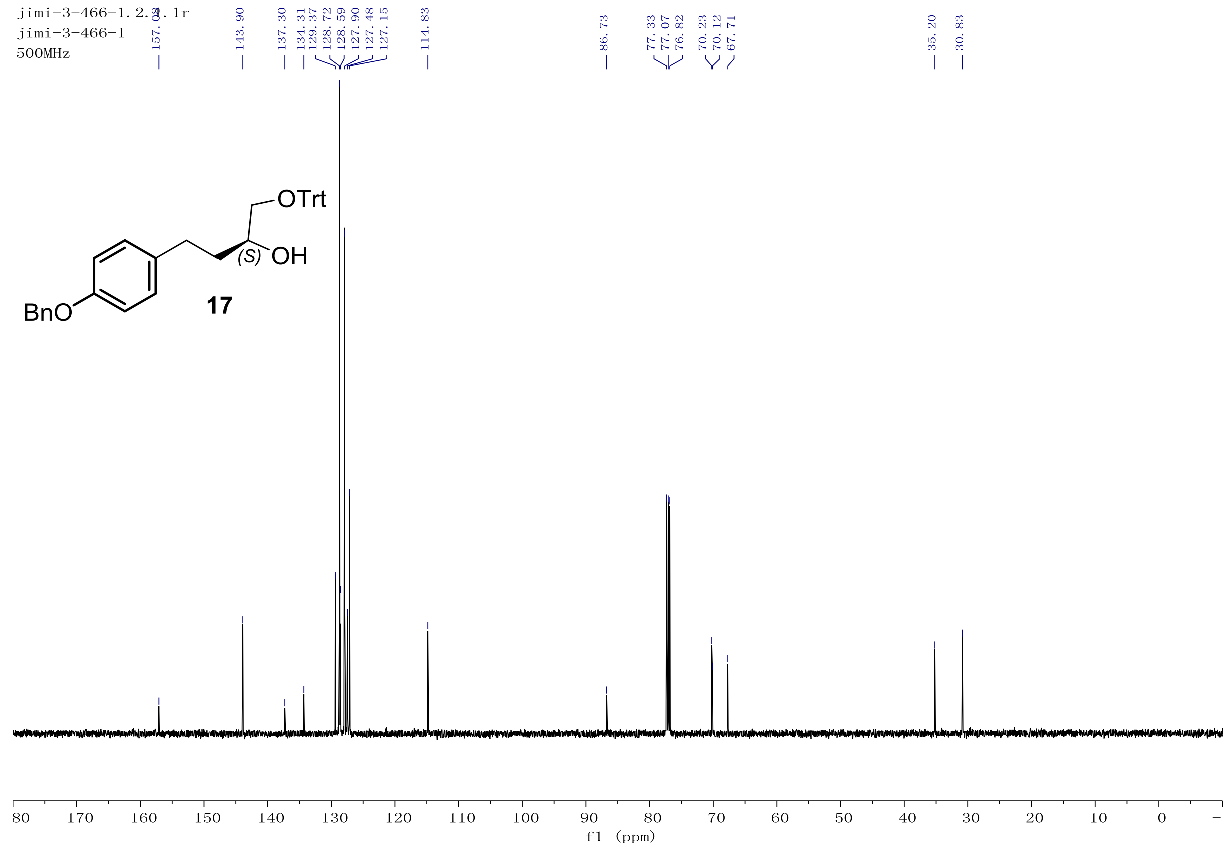

NMR spectra of compound S2 (Bruker 400 MHz, $CDCl_3$)

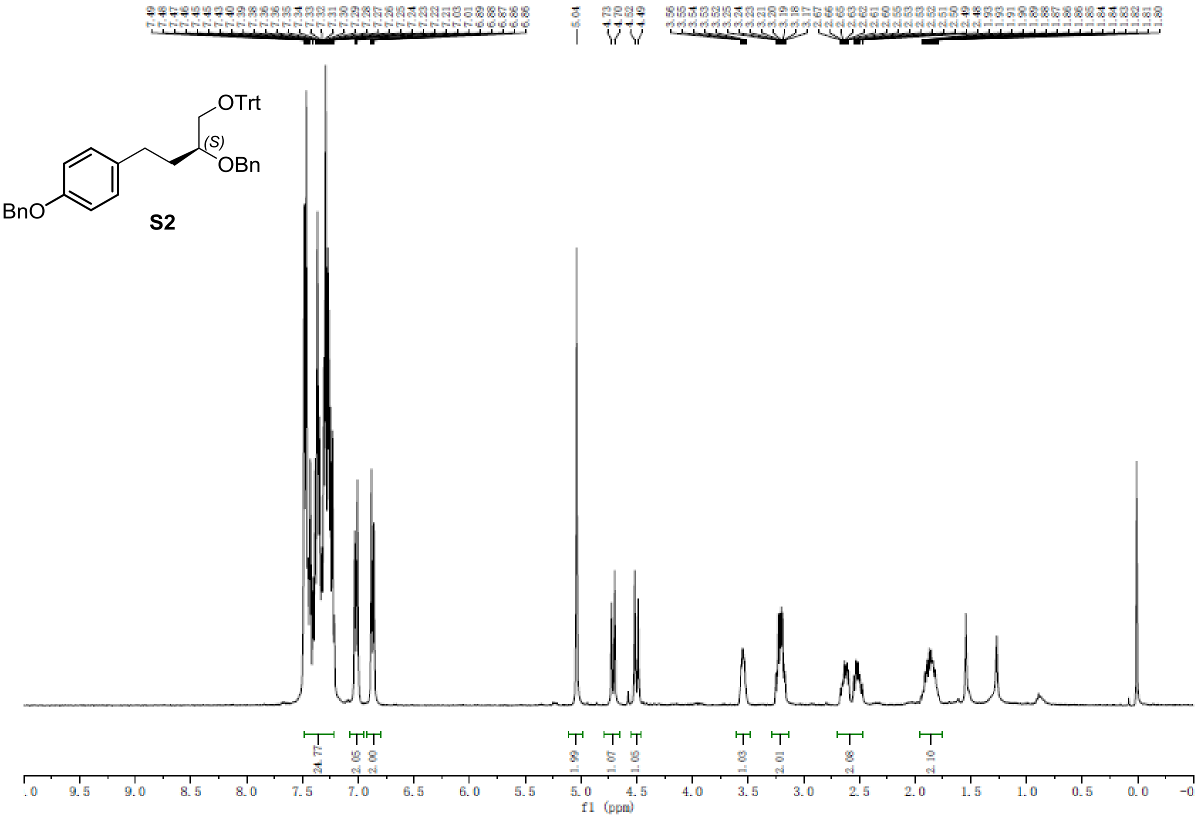

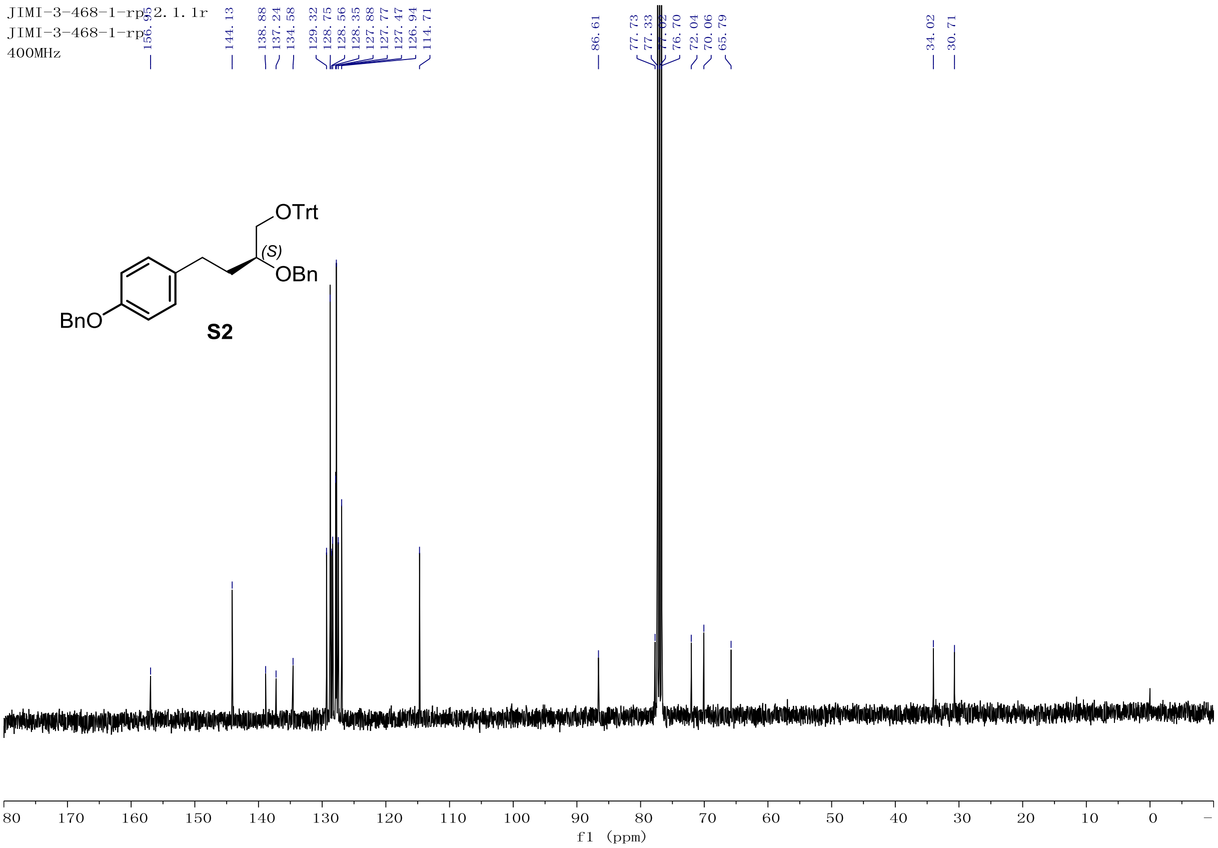

NMR spectra of compound 18 (Bruker 400 MHz, $CDCl_3$)

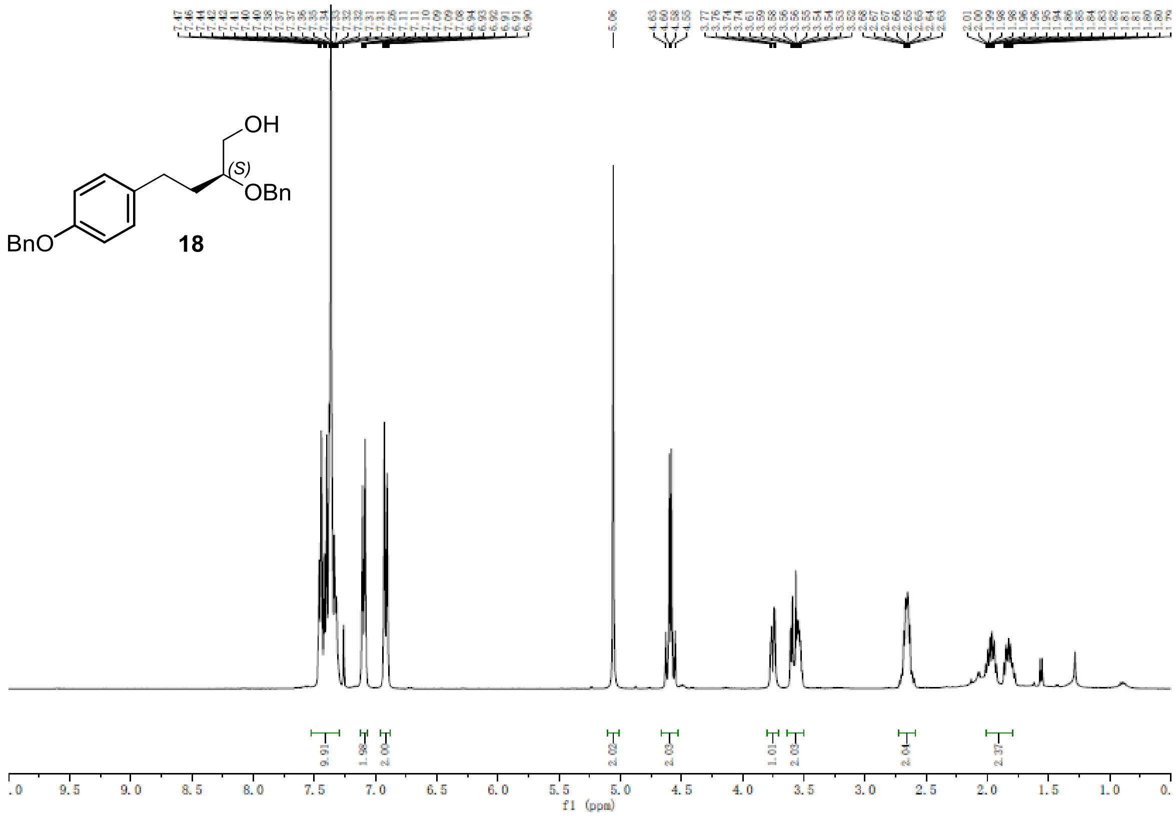

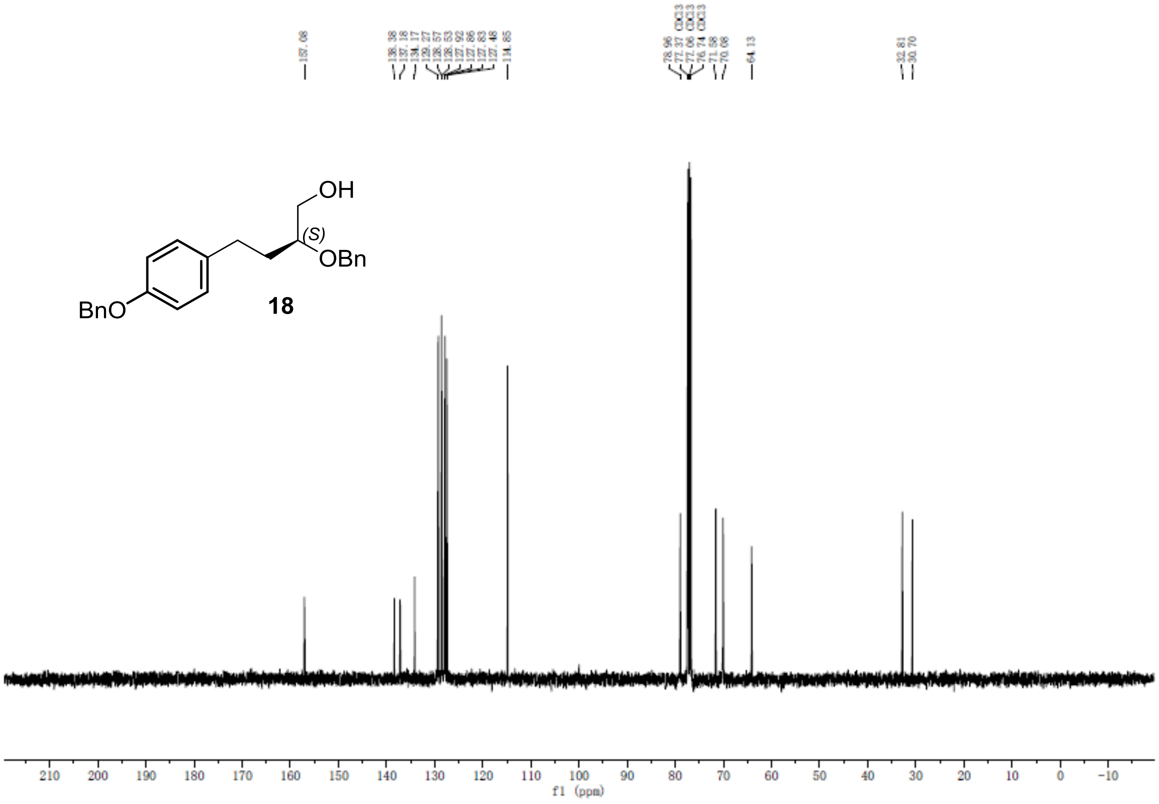

NMR spectra of compound *S*-2 (Bruker 500 MHz, $CDCl_3$)

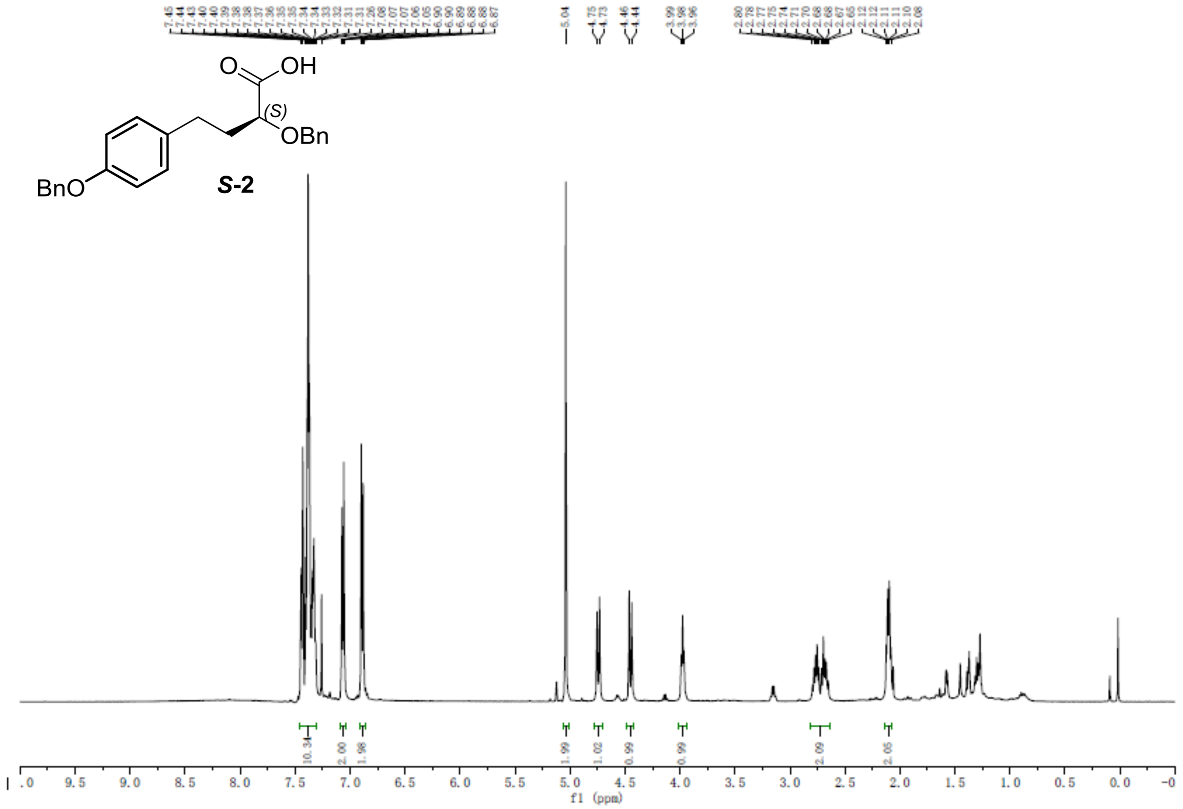

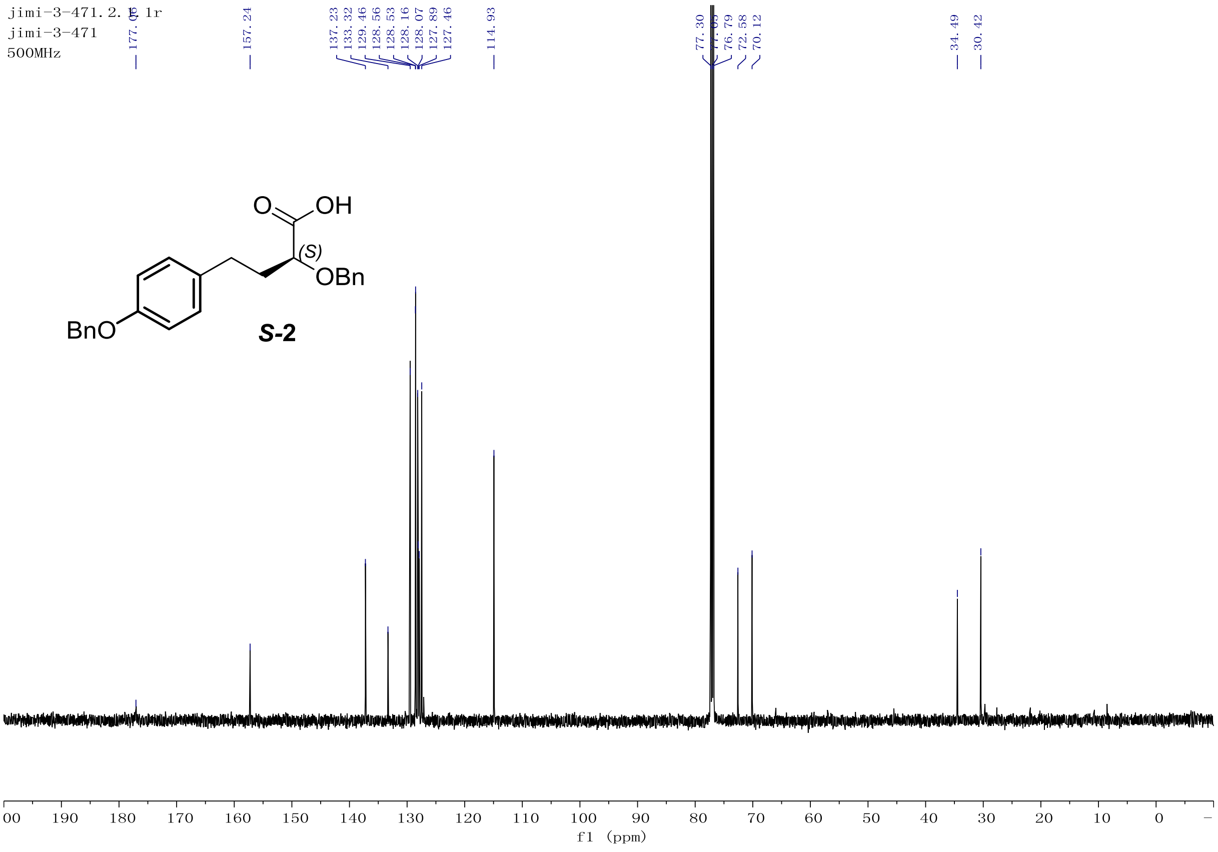

NMR spectra of compound S3 (Bruker 500 MHz, $CDCl_3$)

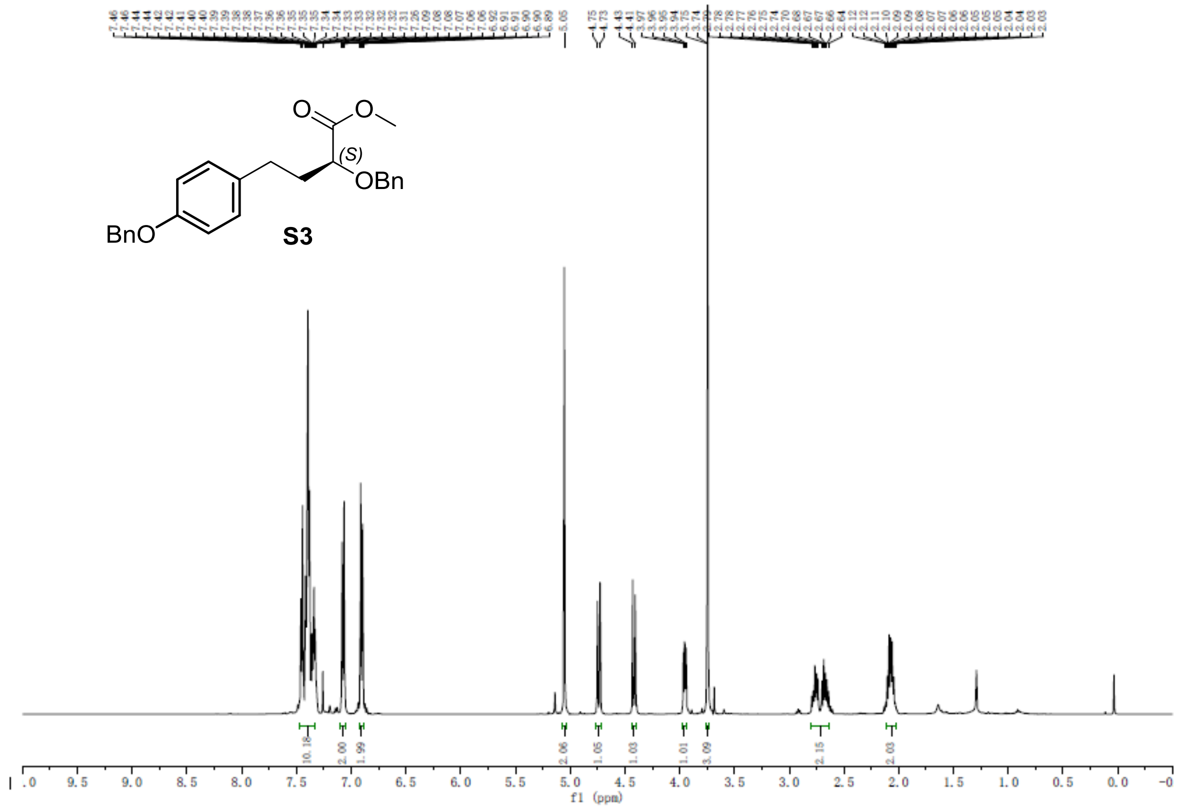

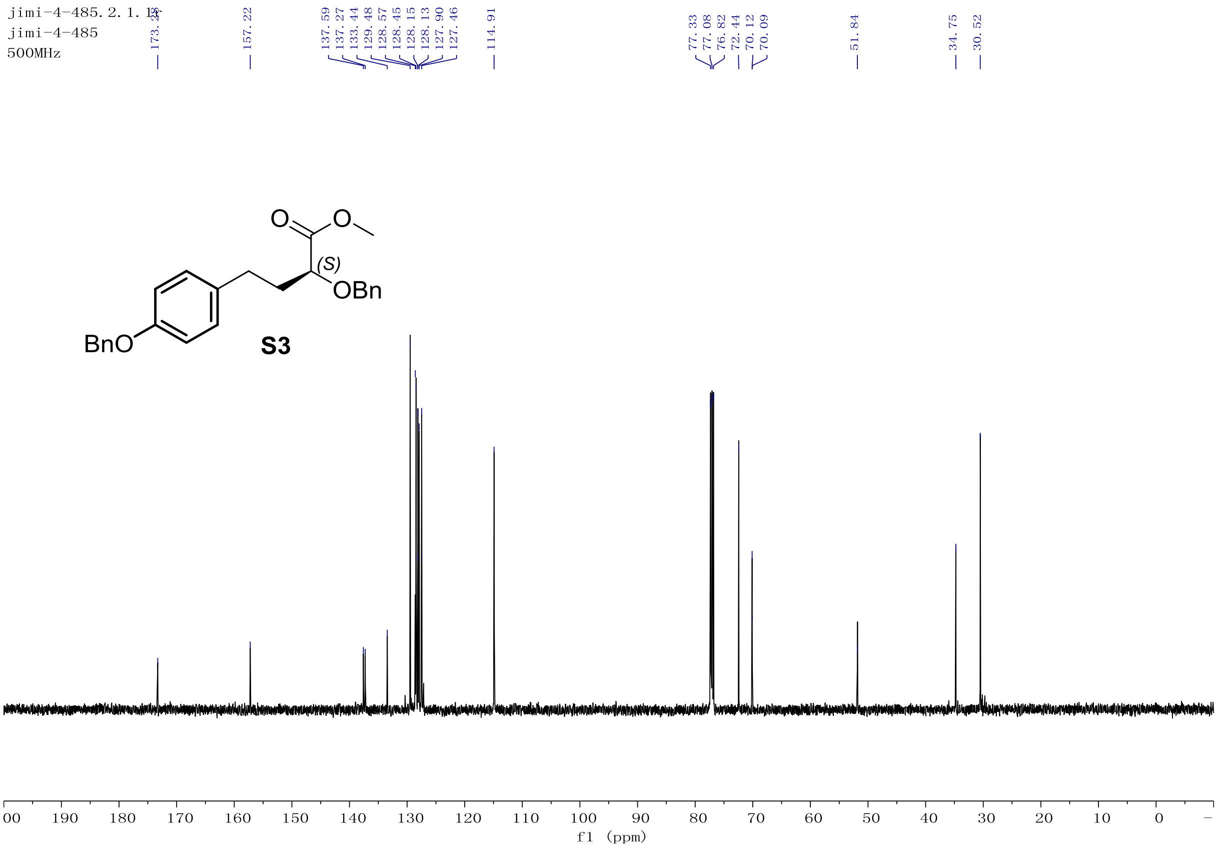

NMR spectra of compound S4 (Bruker 300 MHz, $CDCl_3$)

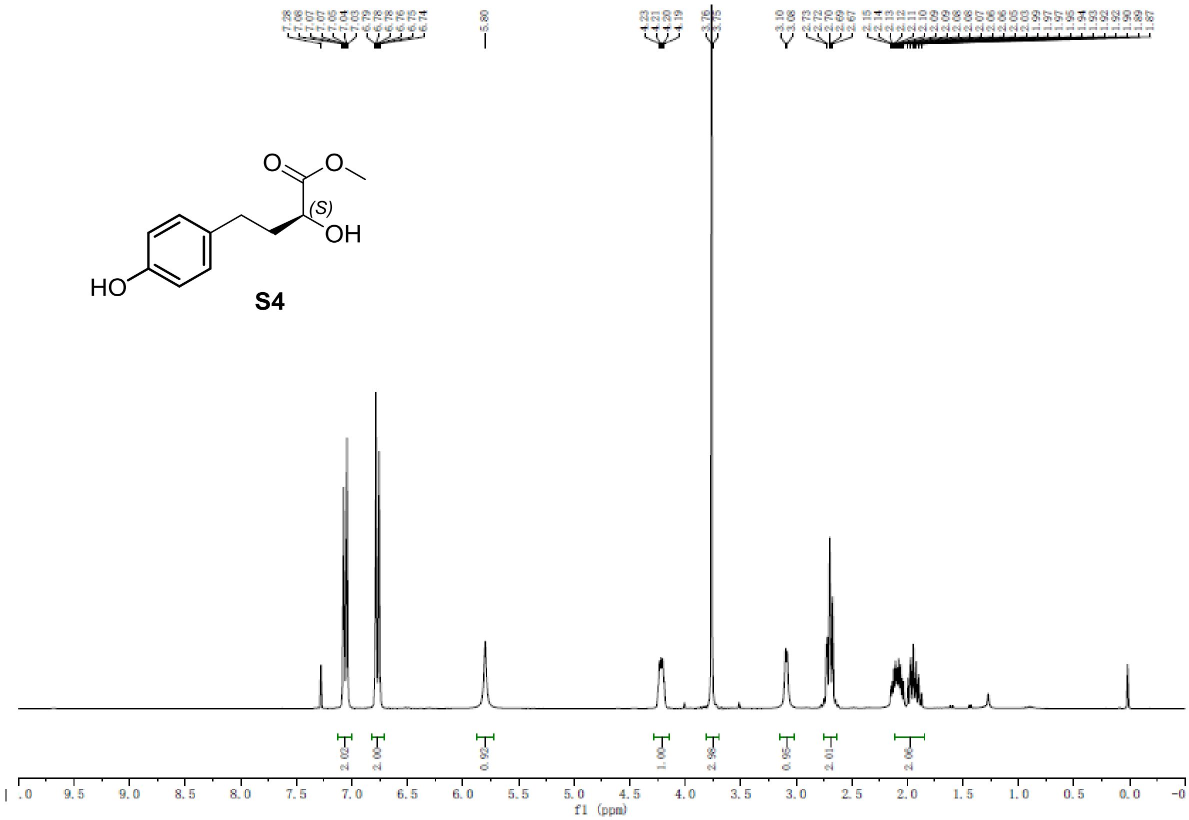

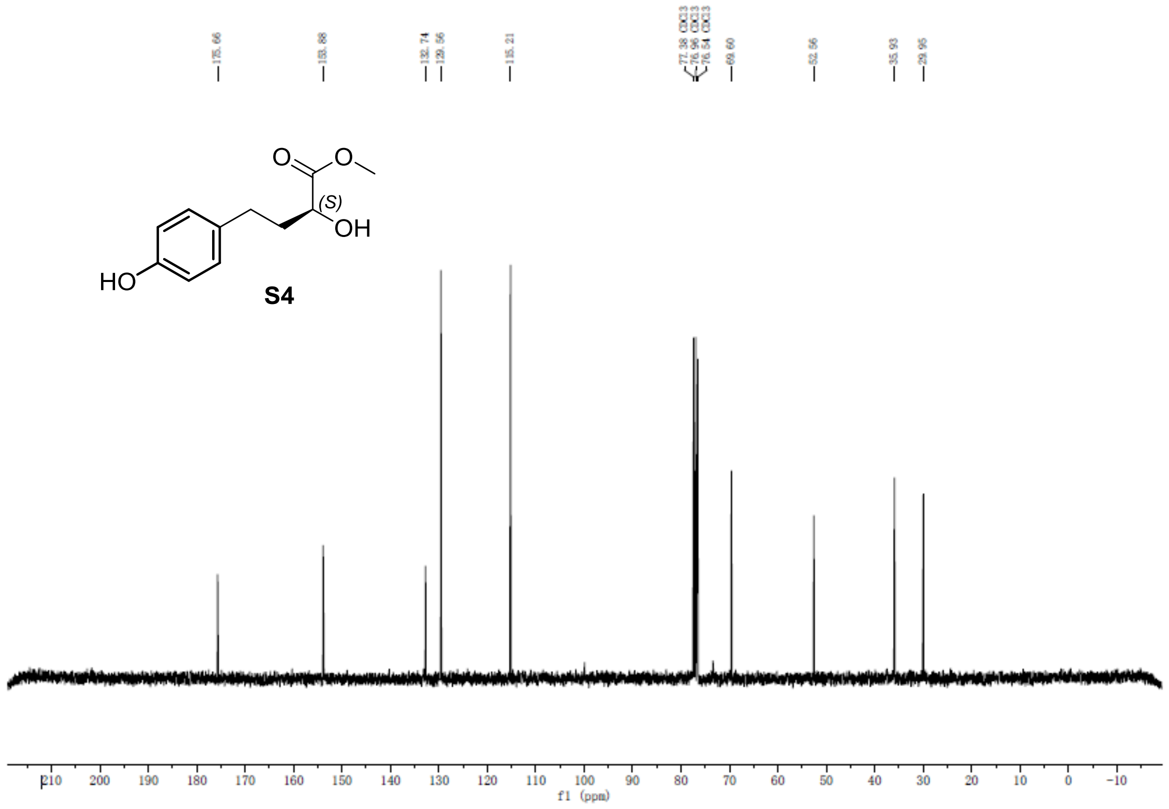

NMR spectra of compound 19 (Bruker 300 MHz, $CDCl_3$)

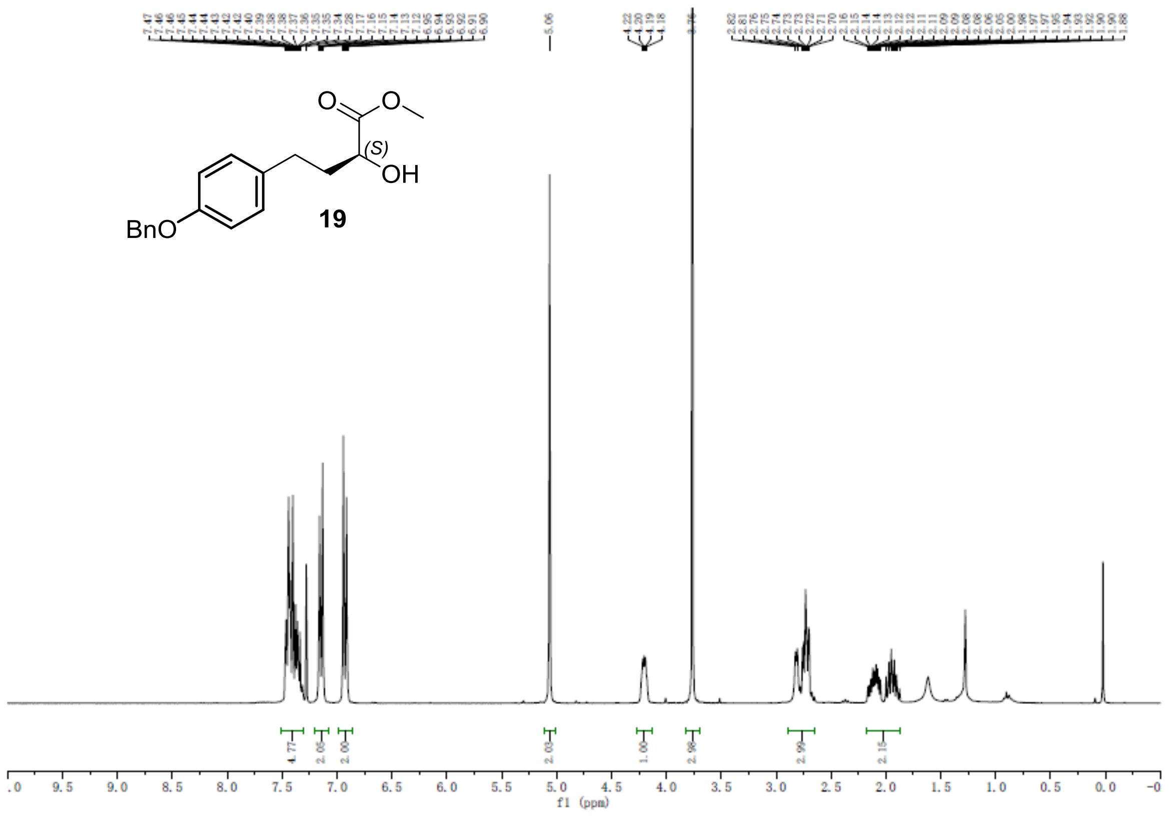

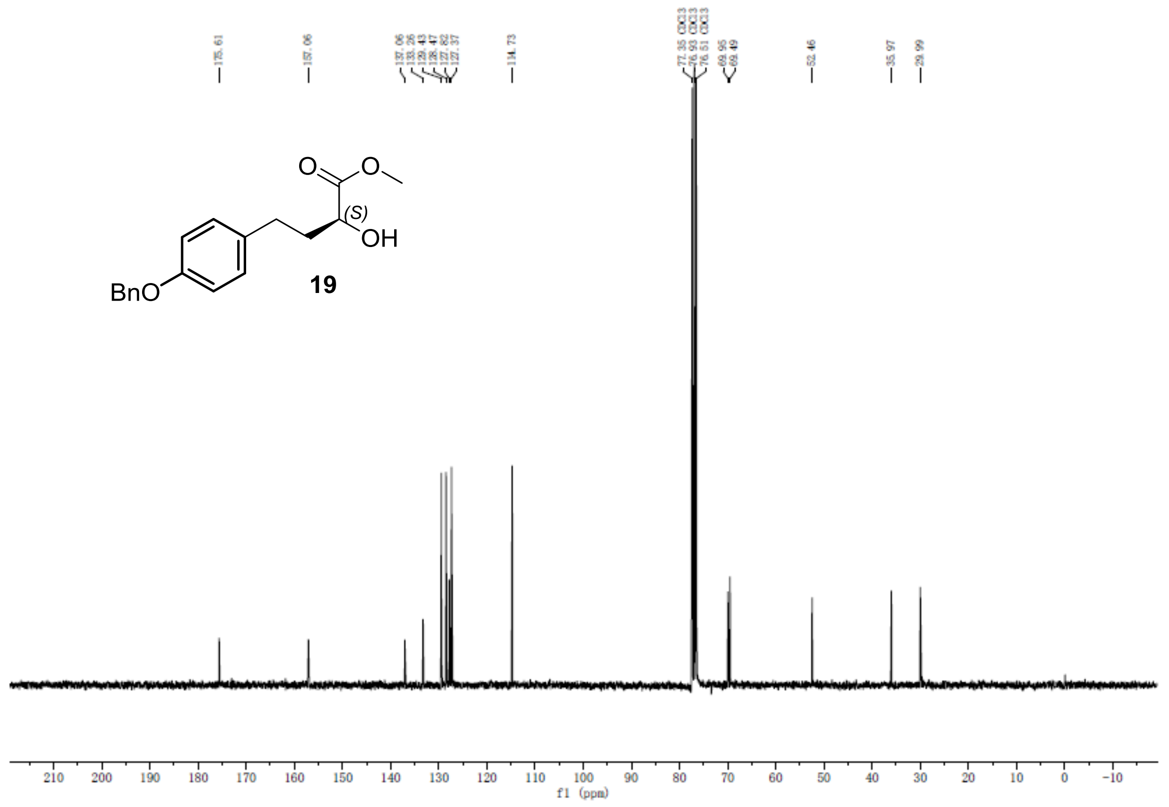

NMR spectra of compound 20 (Bruker 300 MHz, $CDCl_3$)

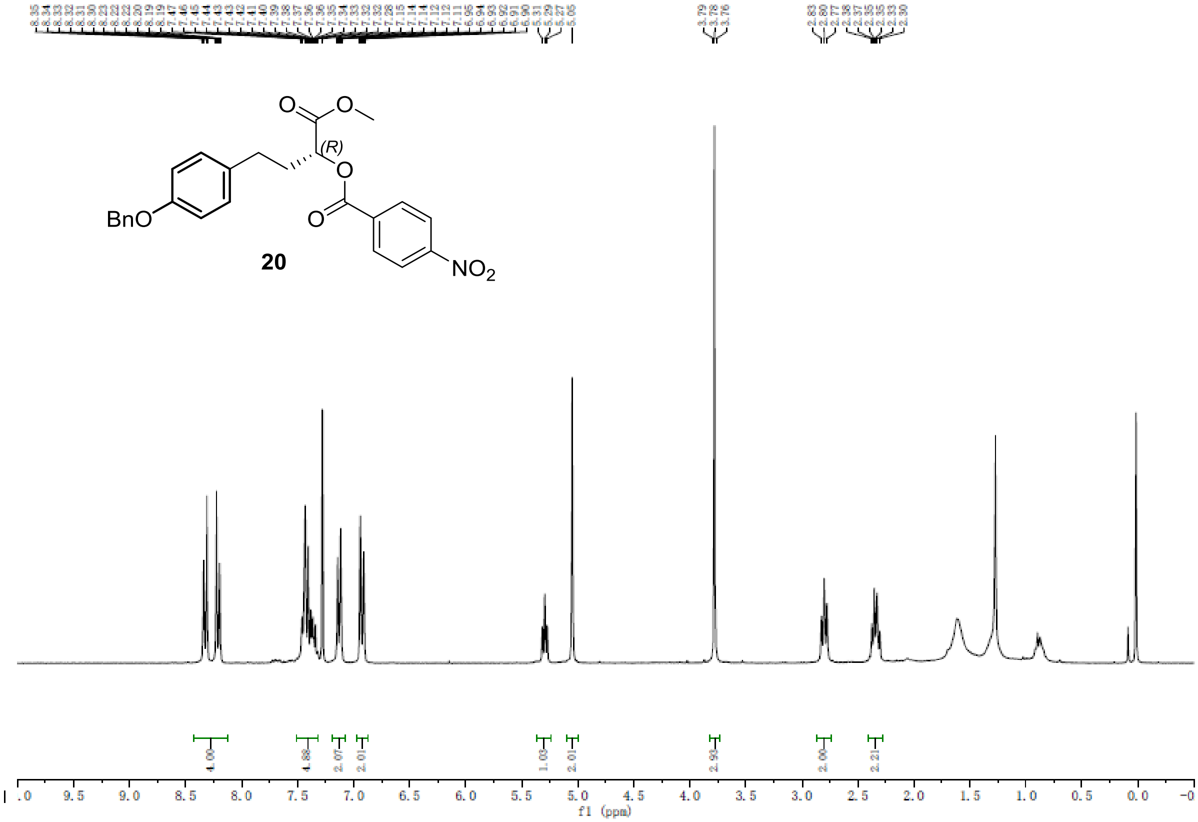

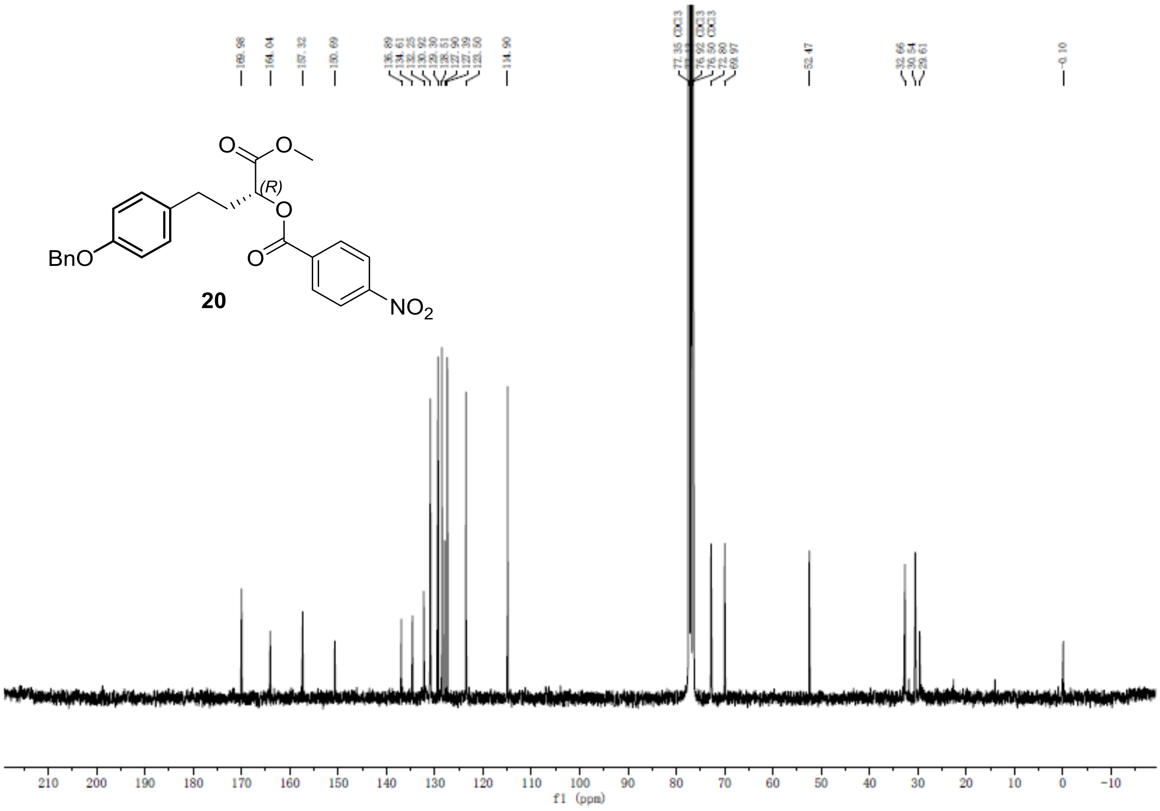

NMR spectra of compound 22 (Bruker 500 MHz, $CDCl_3$)

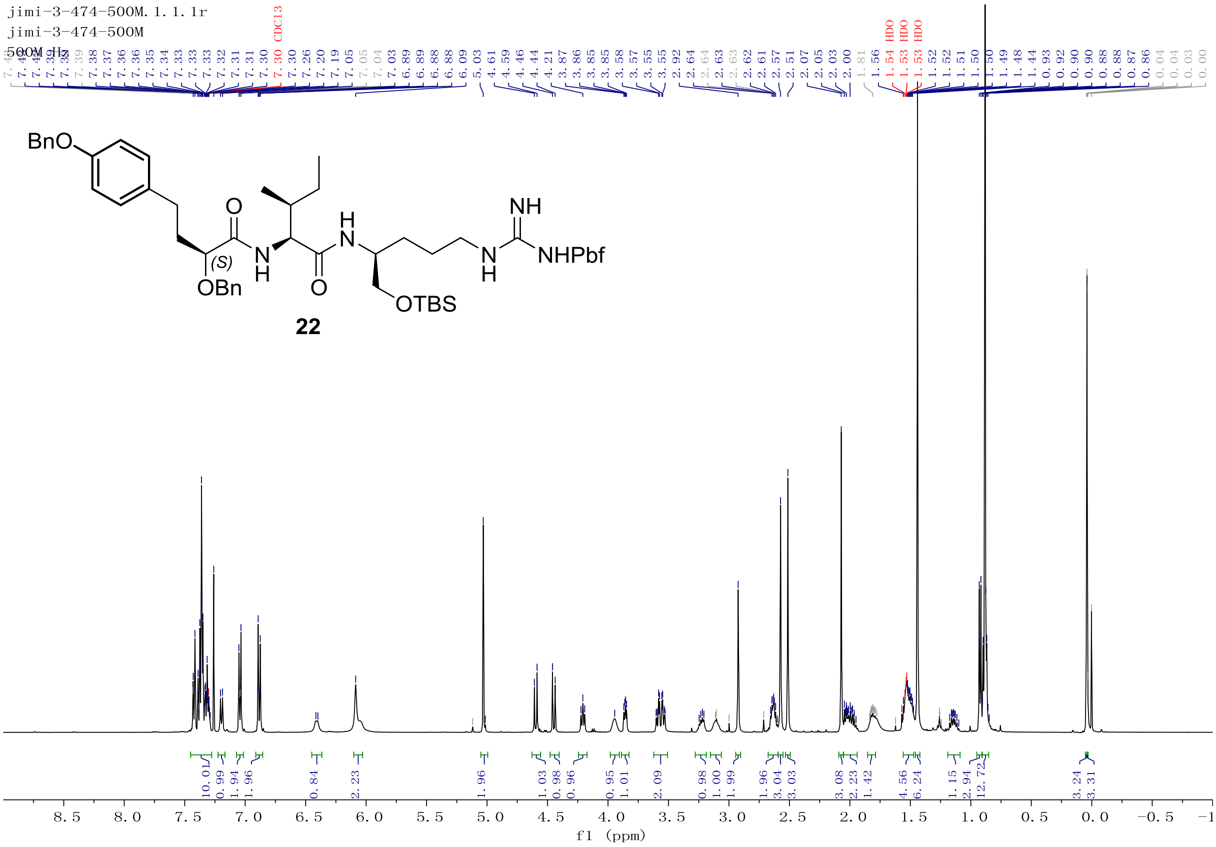

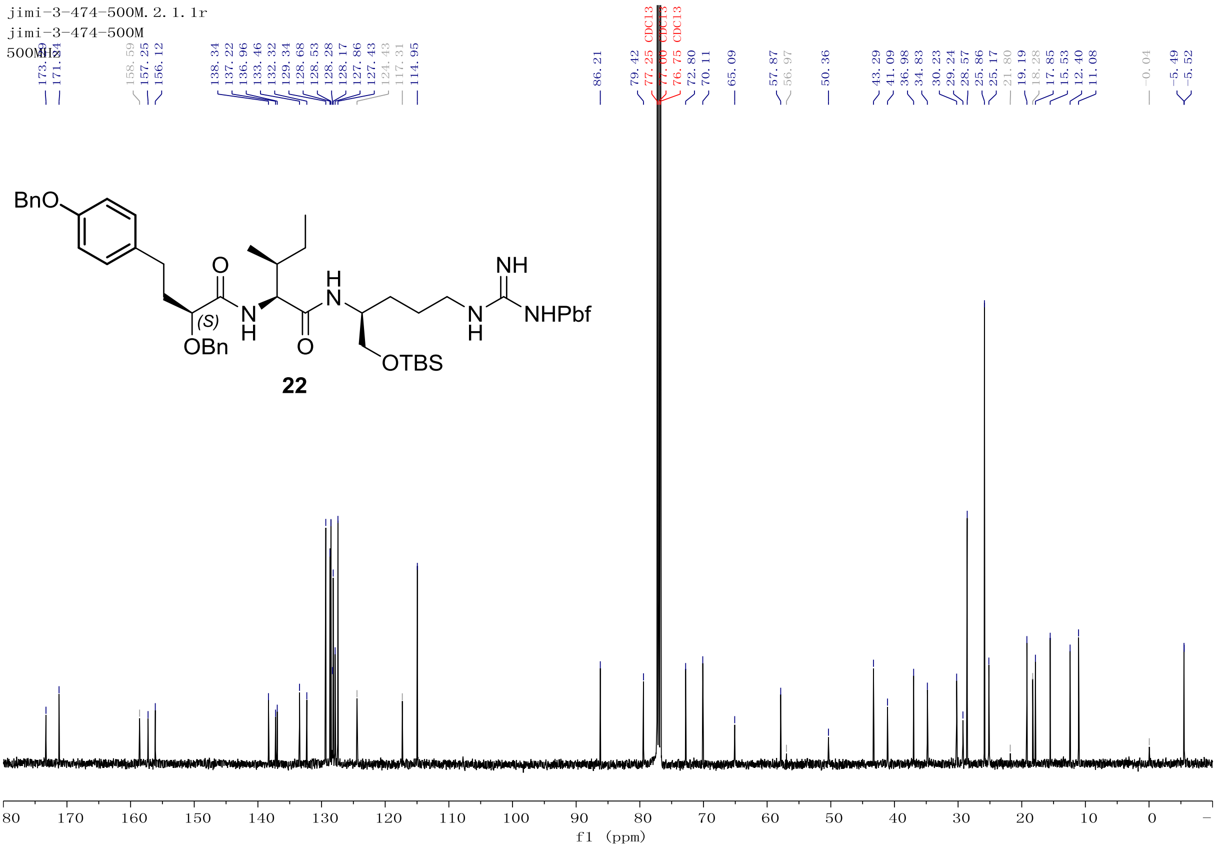

NMR spectra of compound Nostosin B 1a (Bruker 400 MHz, DMSO-d$_6$)

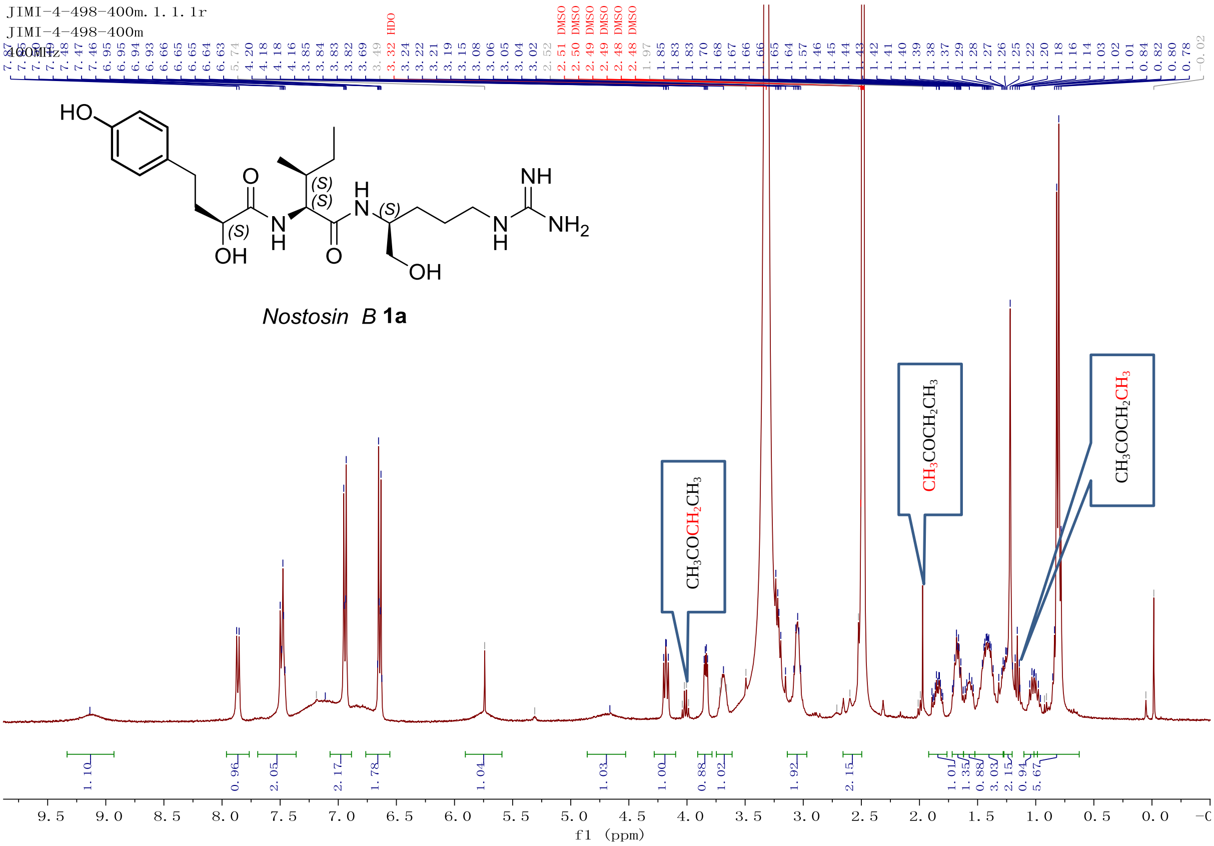

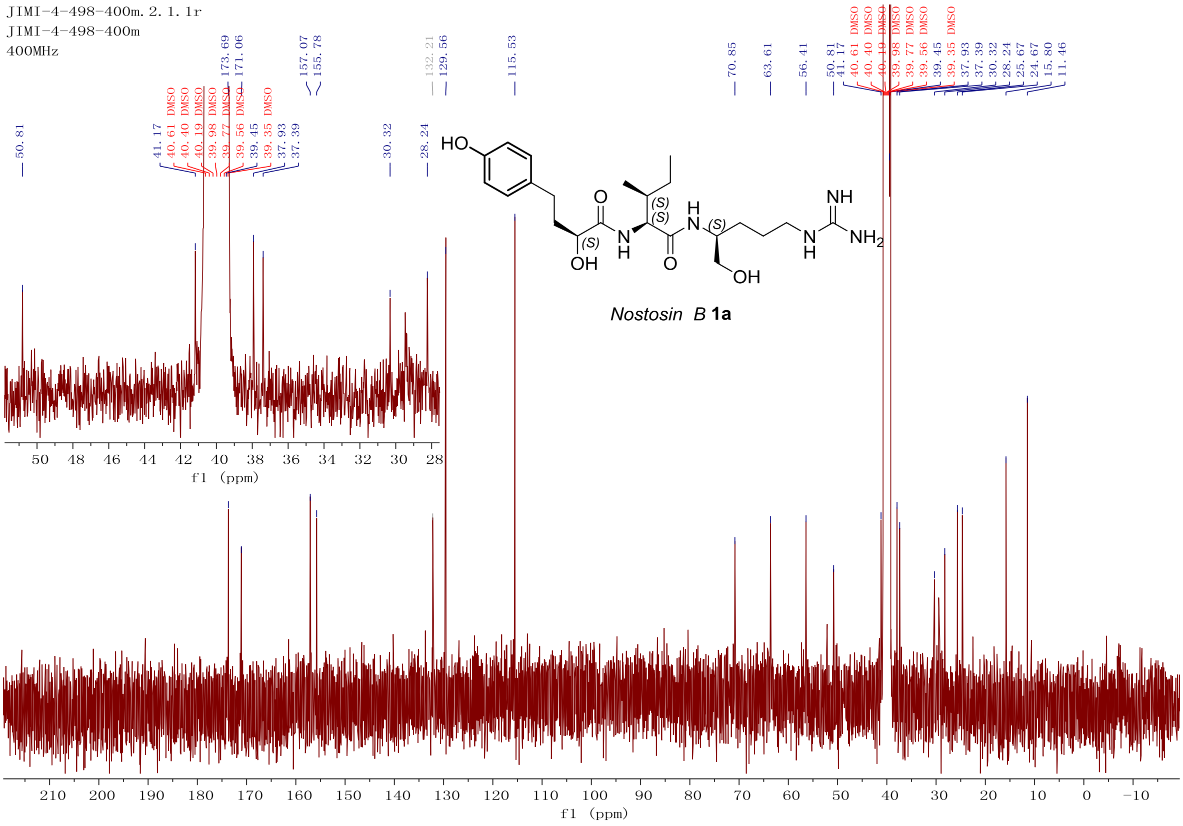

NMR spectra of compound 23 (Bruker 500 MHz, $CDCl_3$)

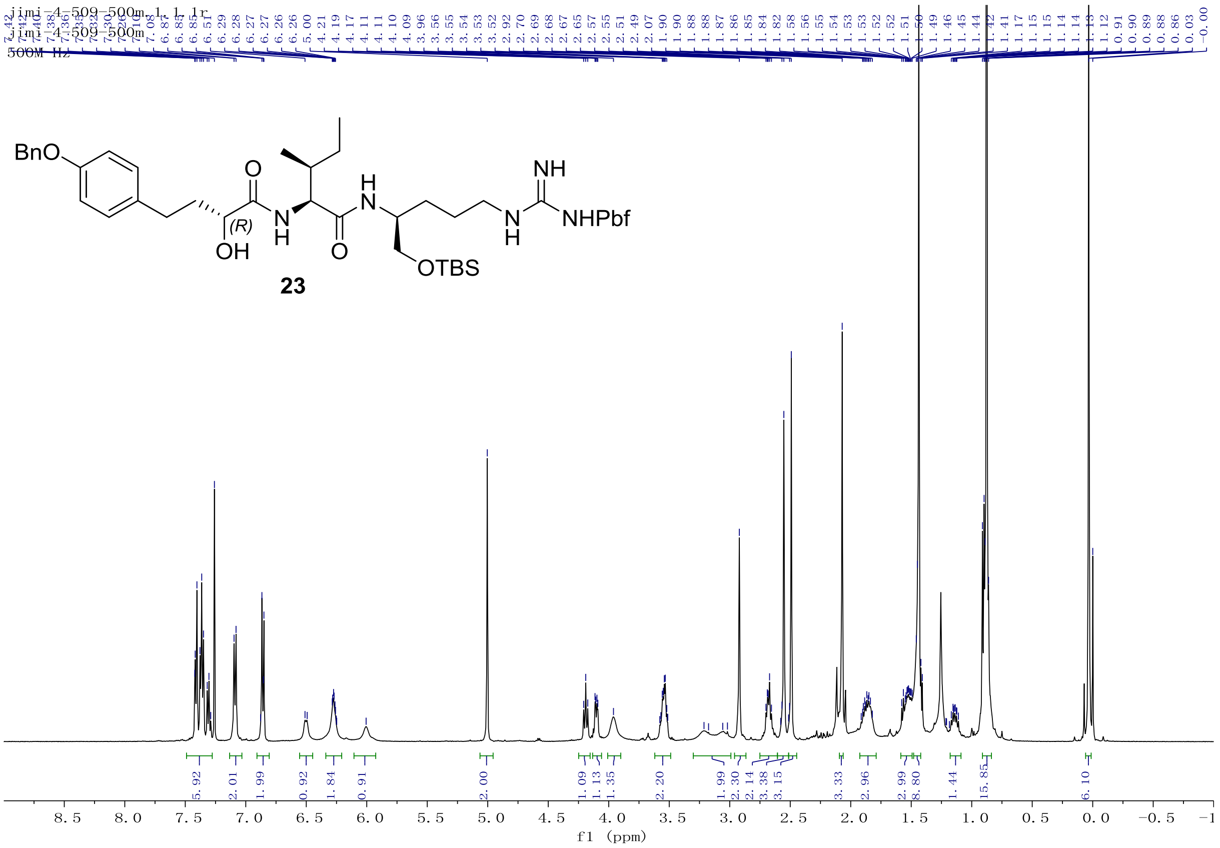

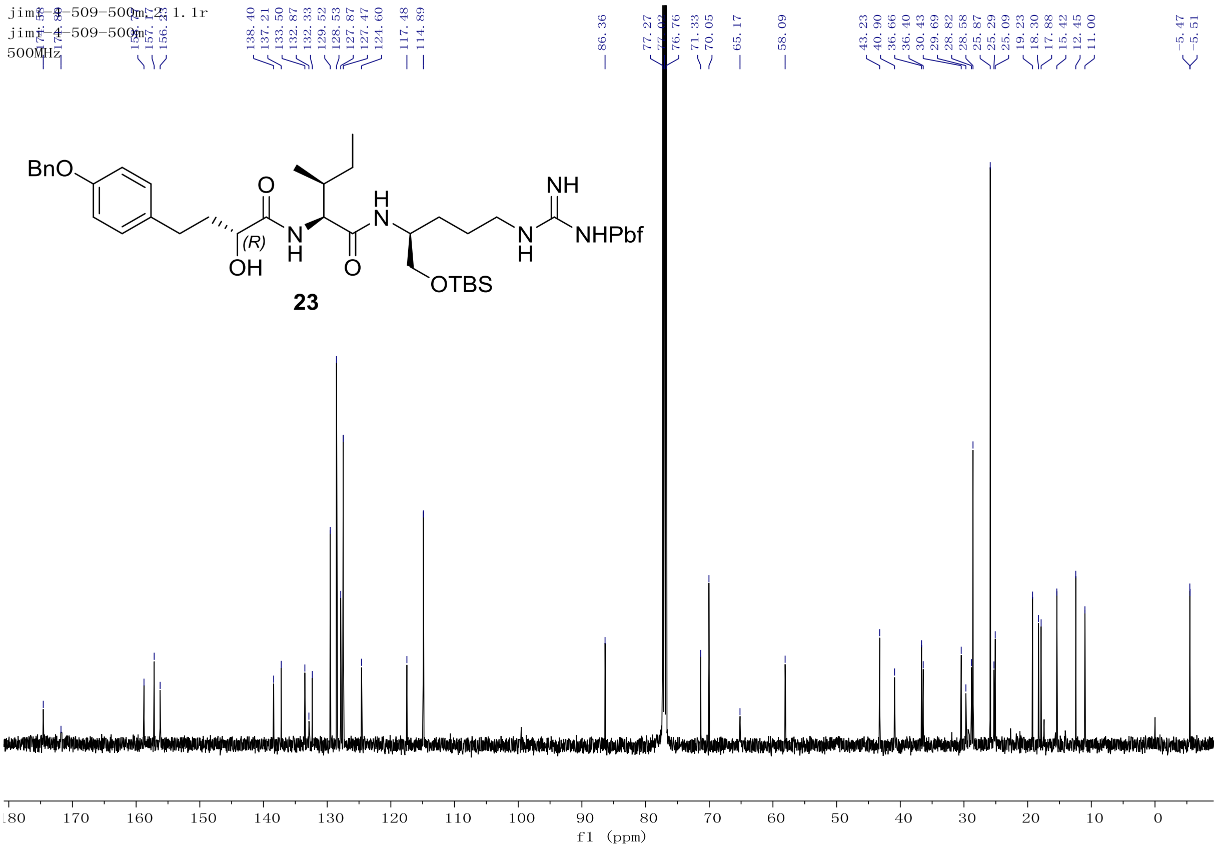

NMR spectra of compound Nostosin B 1b (Bruker 500 MHz, DMSO-d_6)

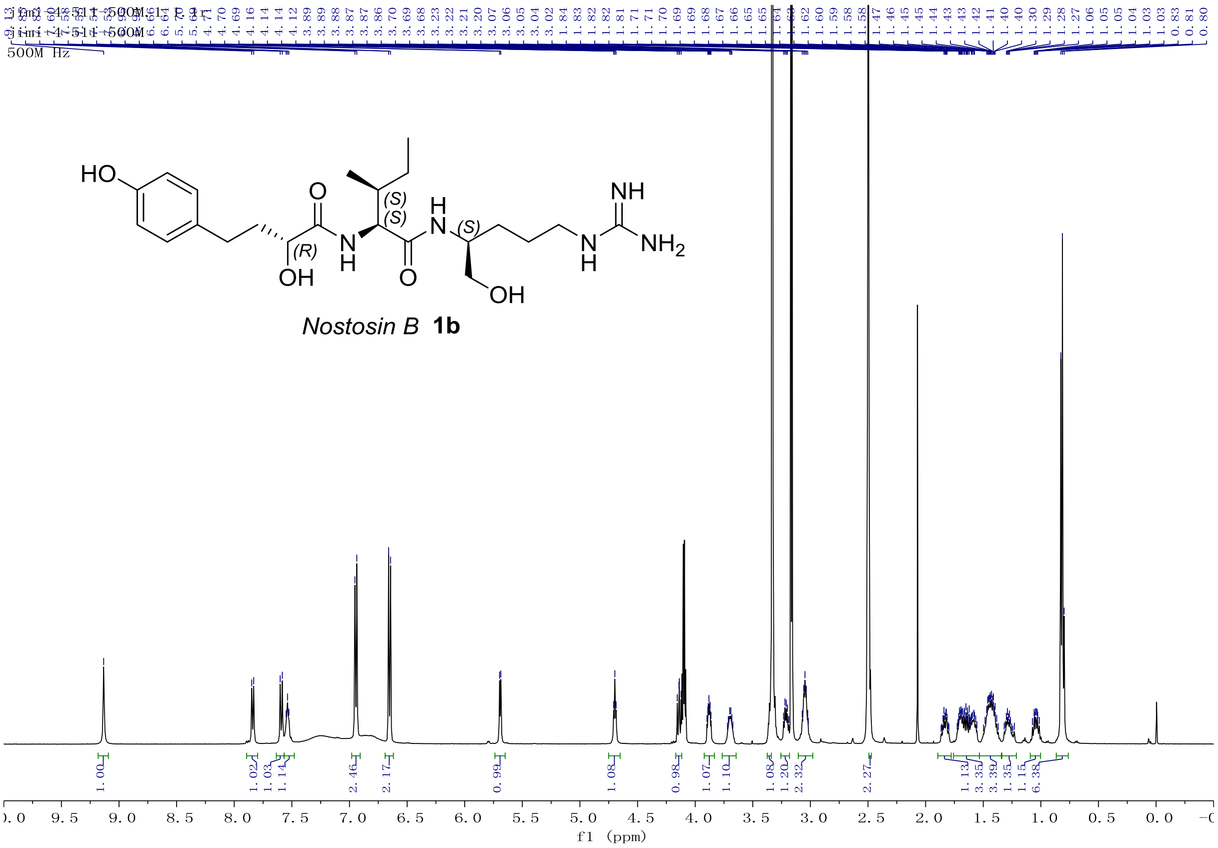

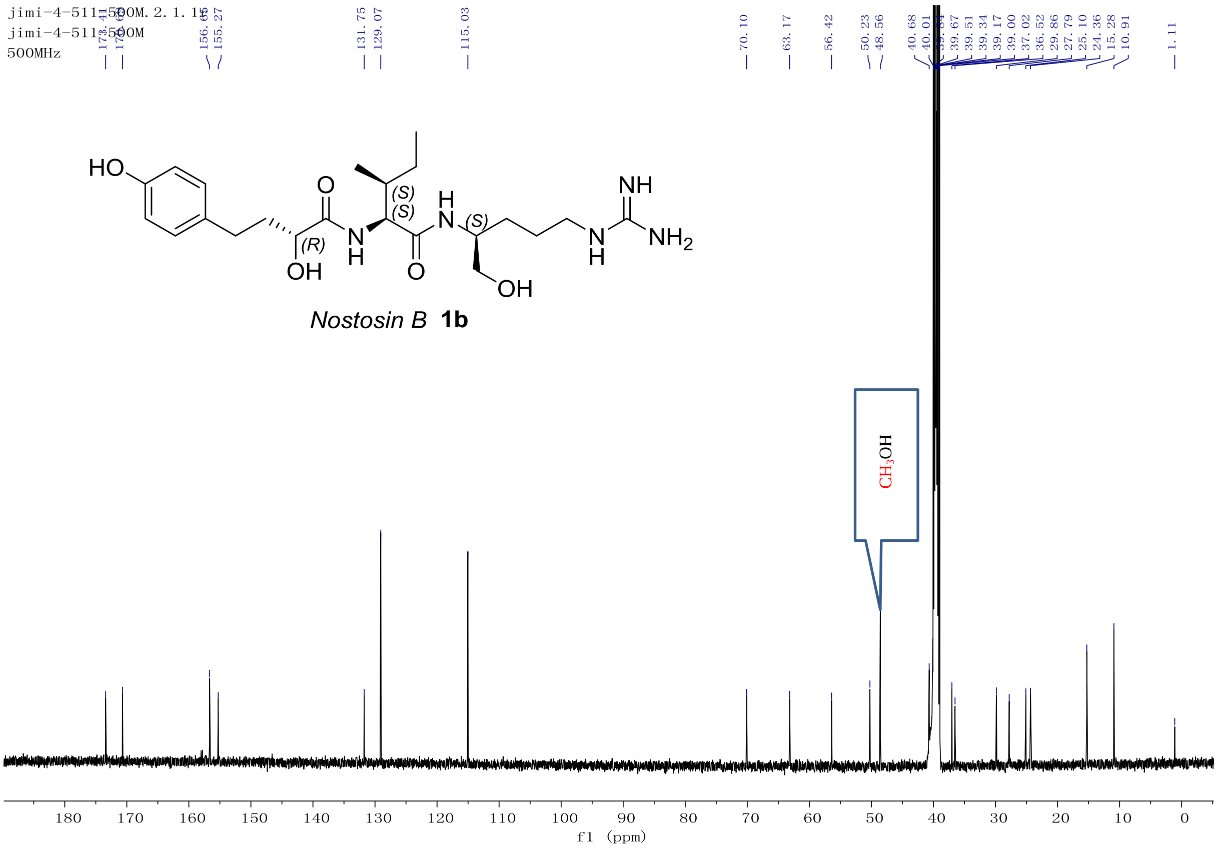

Supplement: Supplementary file 1 [file marinedrugs-15-00058-s001.pdf]
